# High gain DC/DC converter with continuous input current for renewable energy applications

**DOI:** 10.1038/s41598-022-16246-8

**Published:** 2022-07-15

**Authors:** Arafa S. Mansour, AL-Hassan H. Amer, Elwy E. El-Kholy, Mohamed S. Zaky

**Affiliations:** 1 Electrical Engineering Department, Faculty of Engineering, Menoufia University, Shebin Elkom, 32511 Egypt; 2 Department of Electrical Engineering, College of Engineering, Northern Border University, Arar, 1321 Saudi Arabia

**Keywords:** Electrical and electronic engineering, Renewable energy, Solar energy

## Abstract

In this paper, a new design of a non-isolated high-voltage gain DC/DC converter that operates at a reasonable duty cycle, by merging the dual boost converter with the switched inductor structure, is presented as a solution for the high-conversion ratio requirement. The proposed converter operates in discontinuous-current mode (DCM) with zero current switching for all switches and diodes. Wide duty cycle range operation, high output voltage gain, low switching stress, small switching losses, and high efficiency are achieved efficiently. Operating the converter in DCM can support a wide range of the duty cycle operation, maintain lower voltage stress of all devices, ensure the same current sharing among inductors, make it easy to control, provide more stability, and require a smaller inductor which reduces size and weight of the proposed converter. Moreover, the converter operates with a continuous input current. These features make the converter a good choice for many applications such as photovoltaic, x-ray, fuel cells, etc. To prove the converter’s effectiveness, theoretical analysis, project specifications, and operation principles are presented and studied. Experimental results in an open and closed-loop, and a comparison with other recent converters are also introduced to confirm the validity of the proposed converter.

## Introduction

Scientists have been interested in renewable energy sources such as photovoltaic (PV) to produce electricity because they appeared to be the most efficient and effective solution to the environmental problems that the world faces today. The unregulated low-level DC output voltage from these sources is considered the biggest and the most important challenge that requires to be boosted to a regulated higher level using power electronic conditioning. The power electronic interface specification is dependent not only on the renewable energy supply but also on its effects on the power-system operation^1^. To obtain the voltage step-up function, a conventional non-isolated DC/DC boost converter was used^2^ because of its simple structure, simple control, and low cost. But it provided a limited practical gain because of their parasitic elements and must be operated at an extreme duty cycle in order to obtain high voltage gain. That causes a high-semiconductors voltage stress, diode reverse recovery problems, and high switching loss which decrease the system performance and efficiency. In^3^, cascaded boost converter has been successful in solving some of the problems appeared with conventional boost converter as it can attain a reasonable high voltage gain without working at extreme duty cycle, and the voltage stress through the switches remains lower than the voltage across the load. But, it has higher losses, lower efficiency, and electromagnetic interference problems. In switched-capacitor-based converters^4,5^ and switched capacitor/switched inductor-based converters^6–9^ high-voltage gain with small duty cycle, small voltage stress across the switches can be attained, and these converters can be used in a wide range of power. However, they have some problems such as higher losses, lower efficiency, electromagnetic interference problems, and reverse recovery problem.

The use of high-frequency transformers can increase the voltage gain as well as isolation, and then, full-bridge-based topologies can be used^10^, but with limited power capability, higher losses, lower efficiency, and higher cost. Full-bridge, half-bridge, fly back, forward, and push–pull converters are used at various voltage and power levels^11–14^ where isolation is needed. However, they suffer from numerous restrictions which reduce their efficiency, reduce performance in high step-up applications, and make the system more complicated and bulkier. They have also a limited range of increasing the voltage level besides higher voltage stress. Also, a massive turn-off voltage spikes in the power switch is generated due to the leakage inductance, which results in additional voltage stress on the components that require a snubber circuit to clamp the switch voltage resulting in a bigger size and more expensive. A high gain DC/DC converter utilizing coupled inductor and voltage multiplier cell that achieves high gain at a small duty ratio, and low voltage stress across the semiconductor components is presented in^15^. However, it is a hard switching circuit that shortens the life of its components, furthermore, the voltage multiplier cell makes the system bulky and more expensive. Magnetically coupled inductors topologies are presented in^16–18^ which increase the output voltage gain of the converter with transfer energy stored in coupled inductance and decrease the normalized voltage stress across the semiconductors. However, a clamping circuit is needed to prevent switching spikes and recover the leakage energy due to the leakage inductance which produces voltage spikes and ringing.

Recently, several DC/DC converters are presented such as a high voltage gain quasi-Z-source DC/DC converter^19^ that gives a high voltage gain at the low duty cycle and low voltage stress on the semiconductors. However, it works with hard switching making more losses that affect the system performance and efficiency, and it has a limited gain as it is used only for duty cycles less than 0.3. In^20^, a single switch DC/DC converter with non-coupled inductors is used that achieves high voltage gain with high efficiency. The major drawback is it has a large number of passive components that make the system bulkier and more expensive. A high voltage gain p-type DC/DC converter is presented in^21^ that has the advantages of high gain with small duty cycle, continuous input current, common ground, and low voltage stress on semiconductors devices. However, it operates with hard switching and requires a high number of components that making the system larger and more expensive. To improve the voltage gain, a single switch three-Z-network converter is presented in^22^. Although a high voltage gain is achieved, it has a large number of passive components which increase the losses and reduce the efficiency. An impedance network DC/DC boost converter is used in^23^ that reaches a high voltage gain with a small number of diodes and small duty cycle that avoids instability caused by saturation of its inductors. But, the main drawback of the converter is the lower efficiency. In^24^, a step-up DC/DC converter with switched capacitor cells is presented. The converter provides high voltage gain at low duty ratios, low voltage stress on the switches and, switched capacitors, and it can be expanded. However, it has a large number of active and passive components that makes the converter size larger and more expensive.

A single power switch high gain DC/DC converter with advantages of continuous input current, a small number of active components, and low voltage stress across the power switch and diodes is proposed in^25^. But, it is limited power and has a large number of passive components. A non-isolated high gain DC/DC converter for dc micro grid applications with a single switch is presented in^26^, with the advantage of simple control, and low voltage stress across the semiconductor devices. Even so, it operates with hard switching and has a large number of passive components. A transformer-less DC/DC converter based on a coupled inductor and switched capacitor–boosting techniques that increase the voltage gain with a low duty cycle is presented in^27^. Although the voltage stresses across the active components are reduced, it operates with hard switching, has large losses and large number of elements, and hence large size and high expensive. A switched-inductor double power switches high gain DC/DC converter (SL-DS-DC) with higher voltage gain is presented in^28^. However, it has more passive and active components which make the system bulkier and more expensive. A simple control scheme to improve the performance of a quadratic boost converter is presented in^29^. This scheme provides a faster transient response and better noise immunity, but it has a large number of passive components, high losses because of hard switching, low efficiency, large size, and more expensive. High gain-switched boost DC/DC converters contain switched capacitor/switched inductor cells are presented in^30^. The converters have advantages such as high voltage gain at non-extreme duty cycle, low voltage stresses across the switches and output diode, and they can be expanded to give higher voltage gain. To provide higher gain, more cells should be added but this makes the system bulkier and is more expensive. In^31^, a transformer-less high step-up DC/DC converter consisting of an active switched-inductor with quasi-Z-source circuit is offered. High voltage gain at the low duty cycle and high efficiency are achieved. The main drawback is the semiconductors’ components increased by increasing the switched-capacitor cells. A double boost-fly back converter is introduced in^32^, the static gain is increased with the reduction of input current ripple where a combination between two conventional boost-fly back converters with input-parallel and floating output is done. However, if the converter operates with a duty cycle less than 0.5, the input current will be discontinuous with greater ripple. Also, with the increased number of fly back cells more sensors are needed which makes the system bulkier and more expensive.

In this paper, a new design of a non-isolated high-voltage gain DC/DC boost converter operating with a reasonable duty cycle by integrating dual boost converter with switched inductor structure is presented. The proposed converter operates with soft-switching (zero current switching (ZCS) mode for all switches and diodes. High voltage gain, low switching stress, small switching losses, and high efficiency are achieved. The operating modes, steady-state analysis, and design guidelines of the proposed circuit are discussed. Experimental results for the open and closed loops are conducted to verify the validity of the proposed circuit.

## Description and operating modes

**Figure 1:**
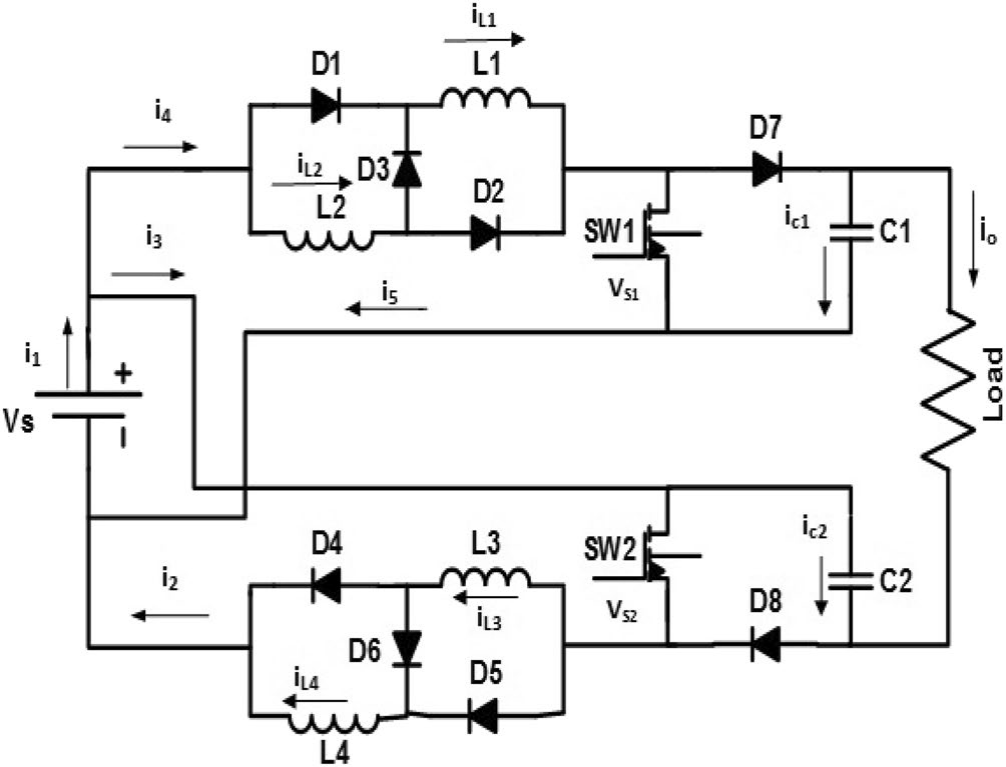
Proposed DC/DC converter.

**Figure 2:**
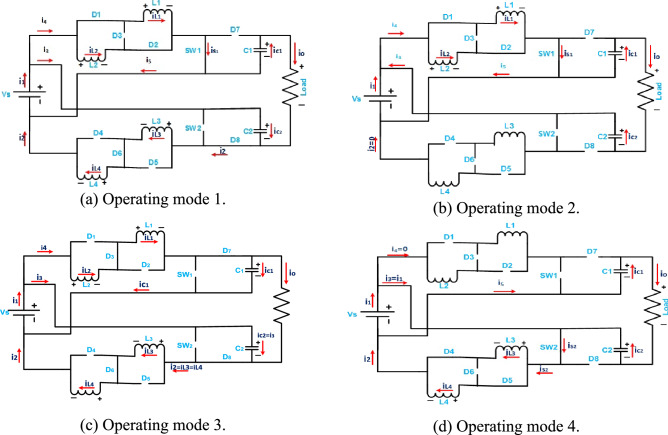
Operating modes of proposed converter. (**a**) Operating mode 1. (**b**) Operating mode 2. (**c**) Operating mode 3. (**d**) Operating mode 4.

**Figure 3:**
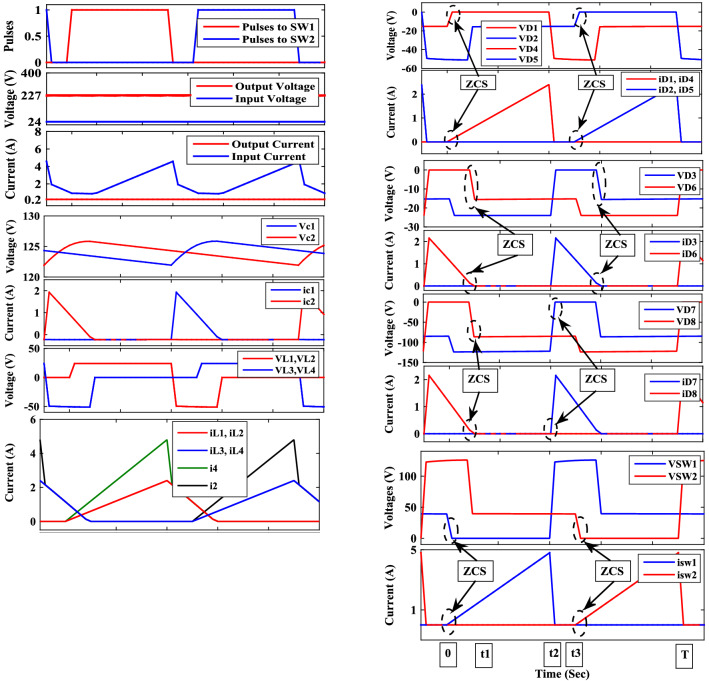
Operating waveforms of the proposed converter at D = 0.4.

The proposed converter is composed of two similar converters connected to the same source as shown in Fig. 1. Each converter has two inductors, one capacitor, four diodes, and one switch. The four inductors have the same magnitude. The two switches are controlled in 180° phase delay to each other simultaneously. The proposed converter works in four modes as presented in Fig. 2. The key operating waveforms of the proposed converter are displayed in Fig. 3.

### Mode 1

In this mode, i.e. (0 ≤ t ≤ t_1_), the switch SW_1_ turns on, the switch SW_2_ turns off, the diodes D_3_, D_4_, D_5_, and D_7_ are reversed biased and the diodes D_1_, D_2_, D_6_, and D_8_ are forward biased. The first switched inductors L_1_ and L_2_ are connected in parallel with each other and being charged through the input voltage source (V_S_) and the current through them increases. The current through the second switched inductors L_3_ and L_4_, which connected in series with each other, decreases and the voltage across them becomes V_S _− V_C2_ that charges the capacitor C_2_ with input source V_S_. However, the capacitor C_1_ discharges through the load. It is noted that V_s_ is in series with both the output capacitors where the output load voltage (V_o_) is V_C1_ + V_C2 _− V_S_.

The voltage and current equations related to this mode are;

1 $$ {\text{V}}_{{\text{S}}} = {\text{V}}_{{{\text{L1}}}} = {\text{V}}_{{{\text{L2}}}} ;{\text{V}}_{{{\text{C2}}}} = {\text{V}}_{{\text{S}}} + {\text{V}}_{{{\text{L3}}}} + {\text{V}}_{{{\text{L4}}}} ;\;{\text{V}}_{{\text{o}}} = {\text{V}}_{{{\text{C1}}}} + {\text{V}}_{{{\text{L3}}}} + {\text{V}}_{{{\text{L4}}}} = {\text{V}}_{{{\text{C1}}}} + {\text{V}}_{{{\text{C2}}}} {-}{\text{V}}_{{\text{S}}} $$

2 $$ {\text{i}}_{{{\text{S1}}}} = {\text{i}}_{{{\text{L1}}}} + {\text{i}}_{{{\text{L2}}}} ;\;{\text{i}}_{{{\text{L3}}}} = {\text{i}}_{{{\text{L4}}}} = {\text{i}}_{{{\text{C2}}}} + {\text{i}}_{{\text{o}}} ;\;{\text{i}}_{{\text{o}}} = {\text{i}}_{{{\text{C1}}}} . $$

### Mode 2

During this mode, i.e. (t_1_ ≤ t ≤ t_2_), the switch SW_1_ is still turn on, the switch SW_2_ is still turn off, the diodes D_3_, D_4_, D_5_, D_6_, D_7_, and D_8_ are reversed biased and the diodes D_1_ and D_2_ are forward biased. The first switched inductors L_1_ and L_2_ are still connected in parallel with each other and being charged through V_S_ and the current through them still increasing. The current through the second switched inductors L_3_ and L_4_ becomes zero as the diodes D_6_ and D_8_ are reversed biased. The capacitors C_1_ and C_2_ discharged through the load, and V_S_ is in series with them.

The voltage and current equations related to this mode are:

3 $$ {\text{V}}_{{\text{S}}} = {\text{V}}_{{{\text{L1}}}} = {\text{V}}_{{{\text{L2}}}} ;\;{\text{V}}_{{\text{o}}} = {\text{V}}_{{{\text{C1}}}} + {\text{V}}_{{{\text{C2}}}} {-}{\text{V}}_{{\text{S}}} $$

4 $$ {\text{i}}_{{{\text{L3}}}} = {\text{i}}_{{{\text{L4}}}} = 0;\;{\text{i}}_{{{\text{S1}}}} = {\text{i}}_{{{\text{L1}}}} + {\text{i}}_{{{\text{L2}}}} ;\;{\text{i}}_{{\text{o}}} = {\text{i}}_{{{\text{C1}}}} = {\text{i}}_{{{\text{C2}}}} . $$

### Mode 3

In this mode, i.e. (t_2_ ≤ t ≤ t_3_), both switches SW_1_ and SW_2_ turn off. The diodes D_1_, D_2_, D_4_, and D_5_ are reversed biased and the diodes D_3_, D_6_, D_7_, and D_8_ are forward biased. The first switched inductors L_1_ and L_2_ are connected in series with each other, and the voltage across them is V_S _− V_C1_. Also, the current through them decreases and charges the capacitor C_1_ with input voltage source V_S_ as diode D_7_ is forward biased. The second switched inductors L_3_ and L_4_ are connected in series with each other, and the voltage across them is V_S _− V_C2_. Also, the current through them decreases and charges the capacitor C_2_ with input voltage source V_S_ as diode D_8_ is forward biased. Both the output capacitors are charged, and their voltage increases. They are in series with V_S_.

The voltage and current equations related to this mode are;

5 $$ {\text{V}}_{{{\text{L1}}}} + {\text{V}}_{{{\text{L2}}}} = \left( { - {\text{V}}_{{{\text{C1}}}} + {\text{V}}_{{\text{S}}} } \right);\;{\text{V}}_{{{\text{L3}}}} + {\text{V}}_{{{\text{L4}}}} = \left( { - {\text{V}}_{{{\text{C2}}}} + {\text{V}}_{{\text{S}}} } \right);\;{\text{V}}_{{\text{o}}} = {\text{V}}_{{{\text{C1}}}} + {\text{V}}_{{{\text{C2}}}} {-}{\text{V}}_{{\text{S}}} $$

6 $$ {\text{i}}_{{1}} = {\text{i}}_{{3}} + {\text{i}}_{{4}} ;\;{\text{i}}_{{3}} = {\text{i}}_{{2}} = {\text{i}}_{{{\text{C2}}}} ;\;{\text{i}}_{{4}} = {\text{i}}_{{{\text{C1}}}} + {\text{i}}_{{\text{o}}} ;\;{\text{i}}_{{1}} = {\text{i}}_{{{\text{C1}}}} + {\text{i}}_{{{\text{C}}2}} + {\text{i}}_{{\text{o}}} . $$

### Mode 4

In this mode, i.e. (t_3_ ≤ t ≤ T_S_), the switch SW_1_ is still turn off and the switch SW_2_ is still turn on. The diodes D_1_, D_2_, D_3_, D_6_, D_7_, and D_8_ are reversed biased, and the diodes D_4_ and D_5_ are forward biased. The current through the first switched inductor L_1_ and L_2_ becomes zero as the diodes D_3_ and D_7_ are reversed biased. The second switched inductors L_3_ and L_4_ are still connected in parallel with each other and being charged through V_S_, and the current through them still increasing. The capacitors C_1_ and C_2_ discharged through the load, and V_s_ is in series with both the output capacitors.

The voltage and current equations related to this mode are;

7 $$ {\text{V}}_{{\text{o}}} = {\text{V}}_{{{\text{C}}1}} + {\text{V}}_{{{\text{C}}2}} {-}{\text{V}}_{{\text{S}}} $$

8 $$ {\text{i}}_{{{\text{L1}}}} = {\text{i}}_{{{\text{L2}}}} = 0;\;{\text{i}}_{{{\text{S2}}}} = {\text{i}}_{{{\text{L3}}}} + {\text{i}}_{{{\text{L4}}}} ;\;{\text{i}}_{{\text{o}}} = {\text{i}}_{{{\text{C1}}}} = {\text{i}}_{{{\text{C2}}}} . $$

### Derivation of voltage gain

For the analysis point of view, the power losses are ignored. For the reason that both converters are boost type, it is assumed that the capacitor voltages V_C1_ and V_C2_ are larger than the input voltage V_S_. Also, both converters have equal duty cycles, the same value of inductances, and the output voltage for each converter has the same average value.

9 $$ {\text{D}}_{{1}} = {\text{D}}_{{2}} = {\text{D}};\;{\text{L}}_{{1}} = {\text{L}}_{{2}} = {\text{L}};\;{\text{V}}_{{{\text{C1}}}} = {\text{V}}_{{{\text{C2}}}} = {\text{V}}_{{\text{C}}} . $$

During the on-state for one converter, the switch SW is closed. Therefore, the input voltage V_S_ appears across the switched inductors. As a result, a change in the current ***Δ***i_L_ flows through the switched inductors during a time period ***Δ***t by the formula: Δ_iL_ = (1/L_eq1_) V_S_ Δt.

$$ \Delta_{{{\text{iL}}}} = (1/{\text{L}}_{{{\text{eq}}1}} ){\text{V}}_{{\text{S}}} \Delta {\text{t}}{.} $$

where L_eq1_ is the equivalent inductance of the switched inductors during the on-state, and it is equal to L/2 as the two inductors are connected in parallel.

At the end of the on-state, the increase of i_L_ is:

$$ \Delta {\text{i}}_{{{\text{L}} - {\text{on}}}} = (1/{\text{L}}_{{{\text{eq}}1}} )\int\limits_{0}^{{{\text{DT}}}} {{\text{V}}_{{{\text{in}}}} } \Delta {\text{t}} = (2/{\text{L}}){\text{V}}_{{\text{S}}} ({\text{DT}} - 0) $$

10 $$ \Delta {\text{i}}_{{\text{L - on}}} = \left( {2{\text{V}}_{{\text{S}}} /{\text{L}}} \right){\text{DT}}{.} $$

Alternatively, the switch SW is open during the off-state. Thus, the inductor current flows through the load. If zero voltage drops in the diode is considered, and the capacitor is large enough for its voltage to stay constant, the evolution of i_L_ is: V_S_ − V_C_ = L (di_L_/ dt).

Then, the change of i_L_ during the off-period is:

$$ \Delta {\text{i}}_{{\text{L - off}}} = (1/{\text{L}}_{{{\text{eq}}2}} )\int\limits_{{{\text{T}}_{{{\text{on}}}} }}^{{\text{T}}} {{\text{V}}_{{{\text{in}}}} } \Delta {\text{t}} $$

where L_eq2_ is the equivalent inductance of the switched inductors during the off-state, and it is equal to 2L as the two inductors are connected in series.

$$ \Delta {\text{i}}_{{\text{L - off}}} = (1/2{\text{L}})\left[ {\int\limits_{{{\text{T}}_{{{\text{on}}}} }}^{{{\text{t}}_{1} }} {( - {\text{V}}_{{{\text{C}}1}} + {\text{V}}_{{\text{S}}} )} \Delta {\text{t}} + \int\limits_{{{\text{t}}_{1} }}^{{\text{T}}} {( - {\text{V}}_{{{\text{SW}}1}} + {\text{V}}_{{\text{S}}} )\Delta {\text{t}}} } \right] $$

and t_1_ = (D + 0.2) T

11 $$ \Delta i_{{\text{L - off}}} = ({\text{T}}/2{\text{L}})[(2 + 14{\text{D}}){\text{V}}_{{\text{S}}} - (2 - 0.5{\text{D}}){\text{V}}_{{{\text{C}}1}} ]. $$

On the basis that the converter works in steady-state conditions, the amount of stored energy in each of its components has to be the same at the beginning and at the ending of the commutation cycle. Particularly, the stored energy in the inductor is given by:

$$ E = 0.{5}\;{\text{L}}\;{\text{i}}^{{2}} . $$

Therefore, the inductor current has to be the same at the start and end of the commutation cycle. This means the overall change in the current (the sum of the changes) is zero:

$$ \Delta {\text{i}}_{{\text{L - on}}} + \Delta {\text{i}}_{{\text{L - off}}} = 0 $$

Substituting Δi_L-on_ and Δi_L-off_ by their expressions yields:

$$ \frac{{{\text{V}}_{C1} }}{{{\text{V}}_{{\text{S}}} }} = \frac{{1 + 9{\text{D}}}}{{1 - 0.25{\text{D}}}} $$

Each module is a separate boost converter with switched inductors. Therefore, the voltage across the output capacitors C_1_ and C_2_ can be expressed as:

12 $$ {\text{V}}_{C1} = {\text{V}}_{{{\text{C}}2}} = \left( {\frac{{1 + 9{\text{D}}}}{{1 - 0.25{\text{D}}}}} \right){\text{V}}_{{\text{S}}} . $$

As clarified through the explanation of the different modes of operation, the two output capacitors are always in series with the input voltage source. Then, the output voltage can be given by:

13 $$ {\text{V}}_{{\text{o}}} = {\text{V}}_{{{\text{C1}}}} + {\text{V}}_{{{\text{C2}}}} {-}{\text{V}}_{{\text{S}}} . $$

From (12) and (13), the voltage gain of the proposed converter can be calculated by:

14 $$ {\text{G}} = \frac{{{\text{V}}_{{\text{o}}} }}{{{\text{V}}_{{\text{S}}} }} = \frac{{1 + 18.25{\text{D}}}}{{1 - 0.25{\text{D}}}}. $$

## Design consideration

In this part, the main converter components are chosen using analytical relations derived from the converter operation. The design of inductors and capacitors depends on both the voltage across them and the current flowing through them.

### Inductor Design

In case of D < 0.5, the inductors and input currents at t = T/2 can be obtained by;

15 $$ {\text{i}}_{4} \left( {\frac{{\text{T}}}{2}} \right) = \left( {\frac{{1 + 9{\text{D}}}}{{1 - 0.25{\text{D}}}}} \right){\text{i}}_{{\text{o}}} + \frac{{\Delta {\text{i}}_{{\text{L}}} }}{2} - \frac{{\Delta {\text{i}}_{{\text{L}}} }}{{1 - {\text{D}}}}(0.5 - {\text{D}});\;{\text{i}}_{2} \left( \frac{T}{2} \right) = \left( {\frac{{1 + 9{\text{D}}}}{{1 - 0.25{\text{D}}}}} \right){\text{i}}_{{\text{o}}} - \frac{{\Delta {\text{i}}_{{\text{L}}} }}{2};\;{\text{i}}_{1} \left( {\frac{{\text{T}}}{2}} \right) = \left( {\frac{{1 + 18.25{\text{D}}}}{{1 - 0.25{\text{D}}}}} \right){\text{i}}_{{\text{o}}} - \frac{{\Delta {\text{i}}_{1} }}{2}. $$

The input current can be obtained by:

16 $$ {\text{i}}_{{1}} = {\text{i}}_{{4}} + {\text{i}}_{{2}} {-}{\text{i}}_{{\text{o}}} . $$

Using (15)–(16), the input current ripple can be expressed as:

17 $$ \Delta {\text{i}}_{1} = \frac{{2(0.5 - {\text{D}})}}{{1 - {\text{D}}}}\Delta {\text{i}}_{{\text{L}}} . $$

The inductor current ripple can be expressed as:

18 $$ \Delta {\text{i}}_{{\text{L}}} = \frac{{({\text{V}}_{{\text{C}}} - {\text{V}}_{{\text{S}}} )(1 - {\text{D}})}}{{{\text{L}}_{{\text{eq - on}}} }}{\text{T}}. $$

From (17) and (18) the inductance can be obtained by:

19 $$ {\text{L}} = \frac{{4\left( {{\text{V}}_{{\text{C}}} - {\text{V}}_{{\text{S}}} } \right)\left( {0.5 - {\text{D}}} \right)}}{{{\text{f}}_{{\text{S}}} \;\Delta {\text{i}}_{1} }} $$

where f_S_ is the switching frequency.

In a similar way in case of D > 0.5, the inductors and input currents at t = T/2 can be obtained by,

20 $$ {\text{i}}_{4} \left( {\frac{{\text{T}}}{2}} \right) = \left( {\frac{{1 + 9{\text{D}}}}{{1 - 0.25{\text{D}}}}} \right){\text{i}}_{{\text{o}}} + \frac{{\Delta {\text{i}}_{{\text{L}}} }}{2} - \frac{{\Delta {\text{i}}_{{\text{L}}} }}{{1 - {\text{D}}}}({\text{D}} - 0.5);\;{\text{i}}_{2} \left( {\frac{{\text{T}}}{2}} \right) = \left( {\frac{{1 + 9{\text{D}}}}{{1 - 0.25{\text{D}}}}} \right){\text{i}}_{{\text{o}}} - \frac{{\Delta {\text{i}}_{{\text{L}}} }}{2};\;{\text{i}}_{1} \left( {\frac{{\text{T}}}{2}} \right) = \left( {\frac{{1 + 18.25{\text{D}}}}{{1 - 0.25{\text{D}}}}} \right){\text{i}}_{o} - \frac{{\Delta {\text{i}}_{1} }}{2}. $$

Using (16) and (20), the input current ripple can be expressed as:

21 $$ \Delta {\text{i}}_{1} = \frac{{2({\text{D}} - 0.5)}}{{1 - {\text{D}}}}\Delta {\text{i}}_{{\text{L}}} . $$

The inductor current ripple can be expressed as:

22 $$ \Delta {\text{i}}_{{\text{L}}} = \frac{{{\text{V}}_{{\text{S}}} (1 - {\text{D}})}}{{{\text{L}}_{{\text{eq - on}}} }}{\text{T}}{.} $$

From (21) and (22) the inductance can be obtained by:

23 $$ {\text{L}} = \frac{{4{\text{V}}_{{\text{S}}} ({\text{D}} - 0.5)}}{{{\text{f}}_{{\text{S}}} \;\Delta {\text{i}}_{1} }}. $$

Finally, the larger value of the two calculated from (19) to (23) is chosen.

### Capacitor Design

In case of D > 0.5, the capacitors and output voltages at t = D.T can be obtained by,

24 $$ \begin{aligned} & {\text{V}}_{{{\text{C}}1}} ({\text{DT}}) = \left( {\frac{{1 + 9{\text{D}}}}{{1 - 0.25{\text{D}}}}} \right){\text{V}}_{{\text{S}}} - \frac{{\Delta {\text{V}}_{{\text{C}}} }}{2};\;{\text{V}}_{{{\text{C}}2}} ({\text{DT}}) = \left( {\frac{{1 + 9{\text{D}}}}{{1 - 0.25{\text{D}}}}} \right){\text{V}}_{{\text{S}}} + \frac{{\Delta {\text{V}}_{{\text{C}}} }}{2} - \frac{{\Delta {\text{V}}_{{\text{C}}} }}{{\text{D}}}({\text{D}} - 0.5); \\ & {\text{V}}_{{\text{o}}} ({\text{DT}}) = \left( {\frac{{1 + 18.25{\text{D}}}}{{1 - 0.25{\text{D}}}}} \right){\text{V}}_{{\text{S}}} - \frac{{\Delta {\text{V}}_{{\text{o}}} }}{2}. \\ \end{aligned} $$

Using (13) and (24), the output voltage ripple can be expressed as:

25 $$ \Delta {\text{V}}_{{\text{o}}} = \frac{{2\left( {{\text{D}} - 0.5} \right)}}{{\text{D}}}\Delta {\text{V}}_{{\text{C}}} . $$

The capacitor voltage ripple can be calculated by:

26 $$ \Delta {\text{V}}_{{\text{C}}} = \frac{{{\text{D}}\;{\text{i}}_{{\text{o}}} }}{{{\text{f}}_{{\text{S}}} \;{\text{C}}}}. $$

From (25) and (26), the capacitance can be obtained by:

27 $$ {\text{C}} = \frac{{2({\text{D}} - 0.5){\text{i}}_{{\text{o}}} }}{{{\text{f}}_{{\text{S}}} \;\Delta {\text{V}}_{{\text{o}}} }}. $$

In a similar way, in case of D < 0.5, capacitor and output voltages at t = D.T can be obtained by,

28 $$ \begin{aligned} & {\text{V}}_{{{\text{C}}1}} ({\text{DT}}) = \left( {\frac{{1 + 9{\text{D}}}}{{1 - 0.25{\text{D}}}}} \right){\text{V}}_{{\text{S}}} - \frac{{\Delta {\text{V}}_{{\text{C}}} }}{2};\;{\text{V}}_{{{\text{C}}2}} ({\text{DT}}) = \left( {\frac{{1 + 9{\text{D}}}}{{1 - 0.25{\text{D}}}}} \right){\text{V}}_{{\text{S}}} + \frac{{\Delta {\text{V}}_{{\text{C}}} }}{2} - \frac{{\Delta {\text{V}}_{{\text{C}}} }}{{\text{D}}}(0.5 - {\text{D}}); \\ & {\text{V}}_{{\text{o}}} ({\text{DT}}) = \left( {\frac{{1 + 18.25{\text{D}}}}{{1 - 0.25{\text{D}}}}} \right){\text{V}}_{{\text{S}}} - \frac{{\Delta {\text{V}}_{{\text{o}}} }}{2}. \\ \end{aligned} $$

Using (13) and (28), the output voltage ripple can be expressed as:

29 $$ \Delta {\text{V}}_{{\text{o}}} = \frac{{2(0.5 - {\text{D}})}}{{\text{D}}}\Delta {\text{V}}_{{\text{C}}} . $$

The capacitor voltage ripple can be expressed as:

$$ \Delta {\text{V}}_{{\text{C}}} = \frac{{{\text{D}}\;{\text{i}}_{{\text{o}}} }}{{{\text{f}}_{{\text{S}}} \;{\text{C}}}}. $$

The capacitance can be obtained by:

30 $$ {\text{C}} = \frac{{2(0.5 - {\text{D}}){\text{i}}_{{\text{o}}} }}{{{\text{f}}_{{\text{S}}} \;\Delta {\text{V}}_{{\text{o}}} }}. $$

Finally, the larger value of the two calculated from (27) and (30) is chosen.

## Converter power losses and efficiency

**Figure 4:**
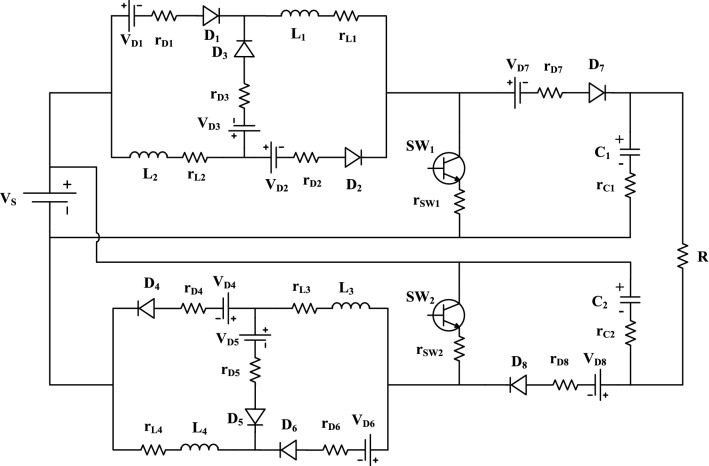
Equivalent model for the proposed circuit with parasitic elements.

In this section, the converter losses are analyzed by calculating the switching losses and conduction losses. The power losses of each device in the proposed converter are estimated, then the total power losses are investigated. Then, the converter efficiency is determined. The proposed converter model with the parasitic components is shown in Fig. 4. For the calculation of conduction loss in the converter, all diodes are considered with cut in voltages *V*_*D*1_, *V*_*D*2_, *V*_*D*3_, *V*_*D*4_, *V*_*D*5_, *V*_*D*6_, *V*_*D*7_ and *V*_*D*8_. Also, the internal resistances are *r*_*D*1_, *r*_*D*2_, *r*_*D*3_, *r*_*D*4_, *r*_*D*5_, *r*_*D*6_, *r*_*D*7_, and *r*_*D*8_. In a Similar way, all inductors and capacitors are also considered with a lumped DC resistance and an equivalent series resistance, respectively. They can be represented as *r*_*L*1_, *r*_*L*2_, *r*_*L*3_, *r*_*L*4_ and *r*_*C*1_, *r*_*C*2_, respectively. Both conduction and switching losses are considered for switch with on-state resistance taken as *r*_*sw*1_, *r*_*sw*2_ for both switches.

### The switches losses

The practical power switch has conduction and switching losses. Assume that the two switches (SW_1_ and SW_2_) have the same *rms* value of current and on-state resistance.

$$ i_{{S{1}}} = i_{{S{2}}} = i_{S} \;{\text{and}}\;r_{{SW{1}}} = r_{{SW{2}}} = {\text{r}}_{SW} $$

The loss of the switching SW_1_ is the sum of the conduction and switching losses and can be written as:

31 $$ P_{loss - total(SW1)} = P_{loss - conduction(SW1)} + P_{loss - switching(SW1)} $$

where the switching loss of SW_1_ can be expressed as:

$$ P_{loss - conduction(SW1)} = i_{Srms}^{2} *r_{SW} $$

32 $$ P_{loss - conduction(SW1)} = \frac{{V_{o}^{2} (1 + 18.25D)^{2} D}}{{4R^{2} (1 - 0.25D)^{2} }}r_{SW} . $$

The switching loss (*P*_loss-switching_) of the power switch SW_1_ can be determined by:

$$ P_{loss - switching(SW1)} = P_{loss - switching(SW1) - on} + P_{loss - switching(SW1) - off} $$

$$ P_{loss - switching(SW1)} = \frac{{t_{on} *V_{SW1} *i_{SW - on} *f_{S} }}{2} + \frac{{t_{off} *V_{SW1} *i_{SW - off} *f_{S} }}{2} $$

33 $$ P_{loss - switching(SW1)} = \frac{{(t_{rt} + t_{ft} )*V_{SW1} *i_{SW1 - avg} *f_{S} }}{2} $$

where *t*_*rt*_ and *t*_*ft*_ is the rise time and fall time of the switch, respectively.

Since the switches operate at ZCS during the turn-on transition period, the switching loss can be expressed as:

$$ P_{loss - switching(SW1)} = \frac{{t_{ft} *V_{SW} *i_{SW - avg} *f_{S} }}{2} $$

34 $$ P_{loss - switching(SW1)} = \frac{{t_{ft} *V_{o}^{2} (1 + 9D)D*f_{S} }}{4R(1 - 0.25D)}. $$

Then, the switching loss of SW_1_ is:

35 $$ P_{loss - total(SW1)} = \frac{{V_{o}^{2} (1 + 18.25D)^{2} D*r_{SW} }}{{4R^{2} (1 - 0.25D)^{2} }} + \frac{{t_{ft} *V_{o}^{2} (1 + 9D)D*f_{S} }}{4R(1 - 0.25D)} $$

and the total switching loss can be expressed as:

$$ P_{loss - total(Switches)} = P_{loss - total(SW1)} + P_{loss - total(SW2)} $$

36 $$ P_{loss - total(Switches)} = \frac{{V_{o}^{2} (1 + 18.25D)^{2} D*r_{SW} }}{{2R^{2} (1 - 0.25D)^{2} }} + \frac{{t_{ft} *V_{o}^{2} (1 + 9D)D*f_{S} }}{2R(1 - 0.25D)}. $$

### The diodes losses

The diodes are assumed to have the same cut in voltages and equivalent series resistance,

$$ \begin{aligned} & V_{{D{1}}} = V_{{D{2}}} = V_{{D{3}}} = V_{{D{4}}} = V_{{D{5}}} = V_{{D{6}}} = V_{{D{7}}} = V_{{D{8}}} = V_{D} \\ & r_{D1} = r_{D2} = r_{D3} = r_{D4} = r_{D5} = r_{D6} = r_{D7} = r_{D8} = r_{D} \\ & i_{D1avg} = i_{D2avg} = i_{D4avg} = i_{D5avg} = i_{Davg} \\ & i_{D1rms} = i_{D2rms} = i_{D4rms} = i_{D5rms} = i_{Drms} \\ & i_{D3avg} = i_{D6avg} = i_{D7avg} = i_{D8avg} = i_{Davg1} \\ & i_{D3rms} = i_{D6rms} = i_{D7rms} = i_{D8rms} = i_{Drms1} . \\ \end{aligned} $$

The total diodes losses can be expressed as:

37 $$ P_{loss - total(Diodes)} = \sum\limits_{i = 1}^{8} {P_{loss - Di} } $$

where

$$ P_{loss - D1} = P_{loss - D2} = P_{loss - D4} = P_{loss - D5} = V_{D} *i_{Davg} + i_{Drms}^{2} *r_{D} $$

$$ P_{loss - D1} = P_{loss - D2} = P_{loss - D4} = P_{loss - D5} = \frac{{V_{D} *V_{o} (1 + 18.25D)D}}{4R(1 - 0.25D)} + \frac{{V_{o}^{2} (1 + 18.25D)^{2} D}}{{16R^{2} (1 - 0.25D)^{2} }}*r_{D} $$

38 $$ P_{loss - D1} = P_{loss - D2} = P_{loss - D4} = P_{loss - D5} = \frac{{V_{o} (1 + 18.25D)D}}{4R(1 - 0.25D)}\left( {V_{D} + \frac{{V_{o} (1 + 18.25D)}}{4R(1 - 0.25D)}*r_{D} } \right) $$

and,

$$ P_{loss - D3} = P_{loss - D6} = P_{loss - D7} = P_{loss - D8} = V_{D} *i_{Davg1} + i_{Drms1}^{2} *r_{D} $$

$$ P_{loss - D3} = P_{loss - D6} = P_{loss - D7} = P_{loss - D8} = \frac{{V_{D} *V_{o} (1 + 18.25D)D}}{2R(1 - 0.25D)} + \frac{{V_{o}^{2} (1 + 18.25D)^{2} D}}{{4R^{2} (1 - 0.25D)^{2} }}*r_{D} $$

39 $$ P_{loss - D3} = P_{loss - D6} = P_{loss - D7} = P_{loss - D8} = \frac{{V_{o} (1 + 18.25D)D}}{4R(1 - 0.25D)}\left( {2V_{D} + \frac{{V_{o} (1 + 18.25D)}}{R(1 - 0.25D)}*r_{D} } \right). $$

Then, the total power loss in the diodes can be determined as:

40 $$ P_{loss - total(Diodes)} = \frac{{V_{o} (1 + 18.25D)D}}{R(1 - 0.25D)}\left( {3V_{D} + \frac{{5V_{o} (1 + 18.25D)}}{4R(1 - 0.25D)}*r_{D} } \right). $$

### The capacitors losses

The proposed converter contains two capacitors. The total power loss due to the two capacitors is given by:

$$ P_{loss - total(Capacitors)} = \sum\limits_{i = 1}^{2} {P_{loss - Ci} } = i_{C1rms}^{2} *r_{C1} + i_{C2rms}^{2} *r_{C2} . $$

The two capacitors are assumed have the same equivalent series resistance, then:

41 $$ P_{loss - total(Capacitors)} = i_{C1rms}^{2} *r_{C1} + i_{C2rms}^{2} *r_{C2} = 2i_{Crms}^{2} *r_{C} . $$

The *rms* value of current through the capacitor can be estimated using the expression:

42 $$ i_{C1rms} = i_{C2rms} = i_{Crms} = \frac{{V_{o} }}{R}\left( {D + \frac{(1 - 0.25D)(1 - D)}{{(1 + 18.25D)}}} \right). $$

Then total power loss due to the two capacitors is given by:

43 $$ P_{loss - total(Capacitors)} = \frac{{2V_{o}^{2} }}{{R^{2} }}\left( {\frac{{0.25(1 + 18.25D)^{2} D}}{{(1 - 0.25D)^{2} }} + \frac{{17(1 - D)D^{2} }}{{(1 - 0.25D)^{2} }} + 0.8(1 - D)} \right)*r_{C} $$

### The inductors losses

The inductors loss can be expressed as

$$ P_{loss - total(Inductors)} = \sum\limits_{i = 1}^{4} {P_{loss - Li} } = \sum\limits_{i = 1}^{4} {i_{Lirms}^{2} *r_{Li} } $$

44 $$ P_{loss - total(Inductors)} = i_{L1rms}^{2} *r_{L1} + i_{L2rms}^{2} *r_{L2} + i_{L3rms}^{2} *r_{L3} + i_{L4rms}^{2} *r_{L4} $$

If the inductors are assumed to have the same internal resistance *r*_*L*1_ = *r*_*L*2_ = *r*_*L*3_ = *r*_*L*4_ = *r*_*L*_, and have the same *rms* value of the inductor currents.

$$ i_{L1rms} = i_{L2rms} = i_{L3rms} = i_{L4rms} = i_{Lrms} $$

$$ P_{loss - total(Inductors)} = i_{Lrms}^{2} (r_{L1} + r_{L2} + r_{L3} + r_{L4} ) $$

45 $$ P_{loss - total(Inductors)} = 4i_{Lrms}^{2} r_{L} . $$

Using the equations in the “Description and operating modes” section, the *rms* value of the inductor current can be noticed

46 $$ i_{Lrms} = \frac{{V_{O} (1 + 18.25D)}}{4R(1 - 0.25D)}(0.4 + 0.6D). $$

Then, substituting in Eq. (45), the total power loss in the inductors can be stated as

47 $$ P_{loss - total(Inductors)} = (\frac{{V_{O}^{2} (1 + 18.25D)^{2} (0.4 + 0.6D)^{2} }}{{4R^{2} (1 - 0.25D)^{2} }}r_{L} . $$

Substituting from Eqs. (36), (40), (43), and (47) into the below equation, the total converter loss can be obtained.

The expression for total losses is as follows:

48 $$ P_{loss - total} = P_{loss - total(Switches)} + P_{loss - total(Diodes)} + P_{loss - total(Capacitors)} + P_{loss - total(Inductors)} . $$

Finally, the efficiency (*ɳ*) of the proposed converter can now be determined as:

49 $$ \eta = \frac{{P_{O} }}{{P_{O} + P_{loss - total} }} $$

where *P*_*o*_ is the output power.

## State space representation

This section presents the state space modelling of the proposed converter. The state variables in the proposed converter are selected as the inductor currents (*i*_*L*1_(*t*), *i*_*L*2_(*t*)), the capacitor voltages (*v*_*C*1_(*t*), *v*_*C*2_(*t*)), and the output voltage (*v*_*Co*_(*t*)). The input variables are chosen as the input voltage (*V*_*s*_(*t*)) and the input current (*i*_*1*_(*t*)). The equivalent circuits showing the converter behavior of the four modes of operation are used as given in Fig. 2. The corresponding state space differential equations are obtained from these equivalent circuits. Kirchhoff’s voltage and current laws are applied for this purpose.

From equivalent circuit of Fig. 2a, the differential equations for operation mode 1 are derived as,

50 $$ \left\{ {\begin{array}{*{20}l} {L_{1} \frac{{di_{L1} (t)}}{dt} = V_{S} } \hfill \\ {L_{2} \frac{{di_{L2} (t)}}{dt} = V_{S} } \hfill \\ {i_{C1} (t) = C_{1} \frac{{dv_{C1} (t)}}{dt} = \frac{vo(t)}{R}} \hfill \\ {i_{C2} (t) = C_{2} \frac{{dv_{C2} (t)}}{dt} = - i_{L1} (t) - i_{L2} (t) + i_{1} (t)} \hfill \\ {\frac{{dv_{o} (t)}}{dt} = \frac{vo(t)}{{RC_{1} }} - \frac{{i_{L1} (t)}}{{C_{2} }} - \frac{{i_{L2} (t)}}{{C_{2} }} + \frac{{i_{1} (t)}}{{C_{2} }}} \hfill \\ \end{array} } \right.. $$

From equivalent circuit of Fig. 2b, the differential equations for operation mode 2 are derived as,

51 $$ \left\{ {\begin{array}{*{20}l} {L_{1} \frac{{di_{L1} (t)}}{dt} = V_{S} } \hfill \\ {L_{2} \frac{{di_{L2} (t)}}{dt} = V_{S} } \hfill \\ {i_{C1} (t) = C_{1} \frac{{dv_{C1} (t)}}{dt} = \frac{{v_{o} (t)}}{R}} \hfill \\ {i_{C2} (t) = C_{2} \frac{{dv_{C2} (t)}}{dt} = \frac{{v_{o} (t)}}{R}} \hfill \\ {\frac{{dv_{o} (t)}}{dt} = \frac{{v_{o} (t)}}{{RC_{1} }} + \frac{{v{}_{o}(t)}}{{RC_{2} }}} \hfill \\ \end{array} .} \right. $$

From equivalent circuit of Fig. 2, the differential equations for operation mode 3 are derived as,

52 $$ \left\{ {\begin{array}{*{20}l} {L_{1} \frac{{di_{L1} (t)}}{dt} = - \frac{{v_{C1} (t)}}{2} + \frac{{V_{S} }}{2}} \hfill \\ {L_{2} \frac{{di_{L2} (t)}}{dt} = - \frac{{v_{C1} (t)}}{2} + \frac{{V_{S} }}{2}} \hfill \\ {i_{C1} (t) = C_{1} \frac{{dv_{C1} (t)}}{dt} = i_{L1} (t) - \frac{{v_{o} (t)}}{R}} \hfill \\ {i_{C2} (t) = C_{2} \frac{{dv_{C2} (t)}}{dt} = i_{1} (t) - i_{L1} (t)} \hfill \\ {\frac{{dv_{o} (t)}}{dt} = \frac{{i_{L1} (t)}}{{C_{1} }} - \frac{{v_{o} (t)}}{{RC_{1} }} - \frac{{i_{L1} (t)}}{{C_{2} }} + \frac{{i_{1} (t)}}{{C_{2} }}} \hfill \\ \end{array} } \right.. $$

From equivalent circuit of Fig. 2d, the differential equations for operation mode 4 are derived as,

53 $$ \left\{ {\begin{array}{*{20}l} {L_{1} \frac{{di_{L1} (t)}}{dt} = 0} \hfill \\ {L_{2} \frac{{di_{L2} (t)}}{dt} = 0} \hfill \\ {i_{C1} (t) = C_{1} \frac{{dv_{C1} (t)}}{dt} = \frac{{v_{o} (t)}}{R}} \hfill \\ {i_{C2} (t) = C_{2} \frac{{dv_{C2} (t)}}{dt} = \frac{{v_{o} (t)}}{R}} \hfill \\ {\frac{{dv_{o} (t)}}{dt} = \frac{{v_{o} (t)}}{{RC_{1} }} + \frac{{v_{o} (t)}}{{RC_{2} }}} \hfill \\ \end{array} } \right.. $$

To derive the transfer function of the proposed converter from the duty ratio to the output voltage, the previous differential equations are used.

The average state space model can be written as:

54 $$ \begin{aligned} & \dot{x}(t) = Ax(t) + Bu(t) \\ & y(t) = Cx(t) \\ \end{aligned} $$

where the coefficient matrices *A*, *B*, and *C* are given by:

55 $$ \begin{aligned} & [A] = A_{1} d_{1} + A_{2} d_{2} + A_{3} d_{3} + A_{4} d_{4} \\ & [B] = B_{1} d_{1} + B_{2} d_{2} + B_{3} d_{3} + B_{4} d_{4} \\ & d = d_{1} + d_{2} + d_{3} + d_{4} = 1 \\ \end{aligned} $$

where

56 $$ \begin{aligned} & d_{1} = \left( {d - 0.5} \right) \\ & d_{2} = \left( {1 - d} \right) \\ & d_{3} = \left( {d - 0.5} \right) \\ & d_{4} = \left( {1 - d} \right). \\ \end{aligned} $$

State space modeling of the proposed DC-DC converter is written as:

57 $$ \begin{aligned} \left[ {\begin{array}{*{20}c} {\dot{i}_{L1} (t)} \\ {\dot{i}_{L2} (t)} \\ {\dot{v}_{C1} (t)} \\ {\dot{v}_{C2} (t)} \\ {\dot{v}_{O} (t)} \\ \end{array} } \right] & = \left[ {\begin{array}{*{20}c} 0 & 0 & {\frac{ - 1}{{L_{1} }}\left( {\frac{2d - 1}{4}} \right)} & 0 & 0 \\ 0 & 0 & {\frac{ - 1}{{L_{1} }}\left( {\frac{2d - 1}{4}} \right)} & 0 & 0 \\ {\frac{1}{{C_{1} }}\left( {\frac{2d - 1}{2}} \right)} & 0 & 0 & 0 & {\frac{2}{{RC_{1} }}\left( {1 - d} \right)} \\ {\frac{ - 1}{{C_{2} }}\left( {2d - 1} \right)} & {\frac{ - 1}{{C_{2} }}\left( {2d - 1} \right)} & 0 & 0 & {\frac{2}{{RC_{2} }}\left( {1 - d} \right)} \\ {\left( {2d - 1} \right)\left( {\frac{ - 1}{{C_{2} }} + \frac{1}{{2C_{1} }}} \right)} & {\frac{ - 1}{{C_{2} }}\left( {2d - 1} \right)} & 0 & 0 & {\left( {1 - d} \right)\left( {\frac{1}{{RC_{1} }} + \frac{1}{{RC_{2} }}} \right)} \\ \end{array} } \right]\left[ \begin{gathered} i_{L1} (t) \hfill \\ i_{L2} (t) \hfill \\ v_{C1} (t) \hfill \\ v_{C2} (t) \hfill \\ v_{O} (t) \hfill \\ \end{gathered} \right] \\ & \quad + \;\left[ {\begin{array}{*{20}c} {\frac{1}{{L_{1} }}\left( {\frac{1 + 2d}{4}} \right)} & 0 \\ {\frac{1}{{L_{2} }}\left( {\frac{1 + 2d}{4}} \right)} & 0 \\ 0 & 0 \\ 0 & {\frac{1}{{C_{2} }}\left( {2d - 1} \right)} \\ 0 & {\frac{1}{{C_{2} }}\left( {2d - 1} \right)} \\ \end{array} } \right]\left[ \begin{gathered} V_{S} (t) \hfill \\ i_{1} (t) \hfill \\ \end{gathered} \right] \\ \end{aligned} $$

58 $$ y(t) = v_{o} (t) = \left[ {\begin{array}{*{20}c} 0 & 0 & 0 & 0 & 1 \\ \end{array} } \right]\left[ \begin{gathered} i_{L1} (t) \hfill \\ i_{L2} (t) \hfill \\ v_{C1} (t) \hfill \\ v_{C2} (t) \hfill \\ v_{O} (t) \hfill \\ \end{gathered} \right]. $$

## Experimental results

**Figure 5:**
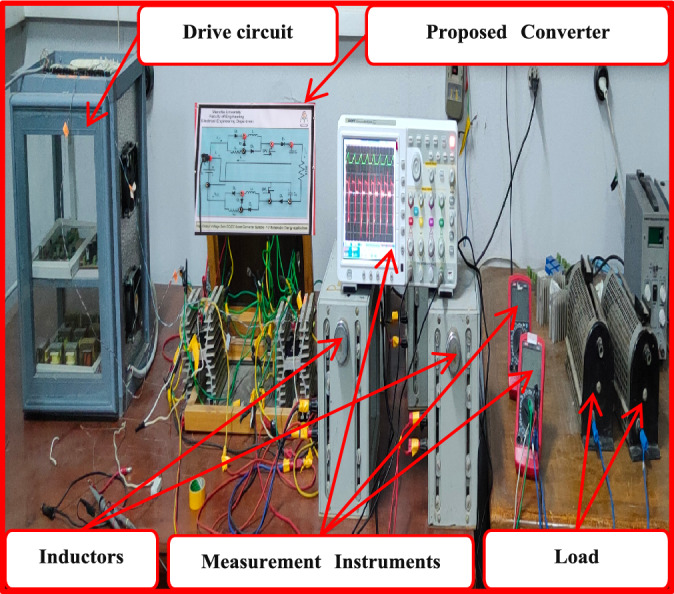
Experimental set up of the proposed converter.

**Figure 6:**
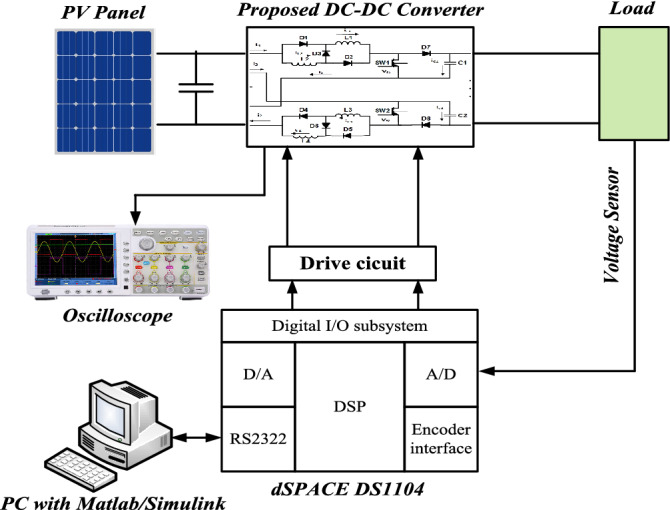
A schematic diagram of the experimental system.

To prove the validity of the proposed converter, it is tested on the laboratory hardware platform as shown in Fig. 5. The schematic diagram of the experimental system is shown in Fig. 6. For experimental analysis, the converter parameters are listed in Table 1.

**Table 1:** Converter component specifications for experimental test.

Devices	Value
Inductances (L_1_, L_2_, L_3_, L_4_)	9.3 mH
Capacitances (C_1_, C_2_)	4.7 μF
Power switches (SW_1_, SW_2_(	CM100DY-24H
Diodes (D_1_, D_2_, D_3_, D_4_, D_5_, D_6_, D_7_, D_8_)	MUR1560
Input voltage (V_S_)	24 V
Output voltage (V_O_)	226 V
Switching frequency (*f*_S_)	1 kHz
Duty cycle (D)	0.4
Gain (G)	9.4

### Open loop performance

**Figure 7:**
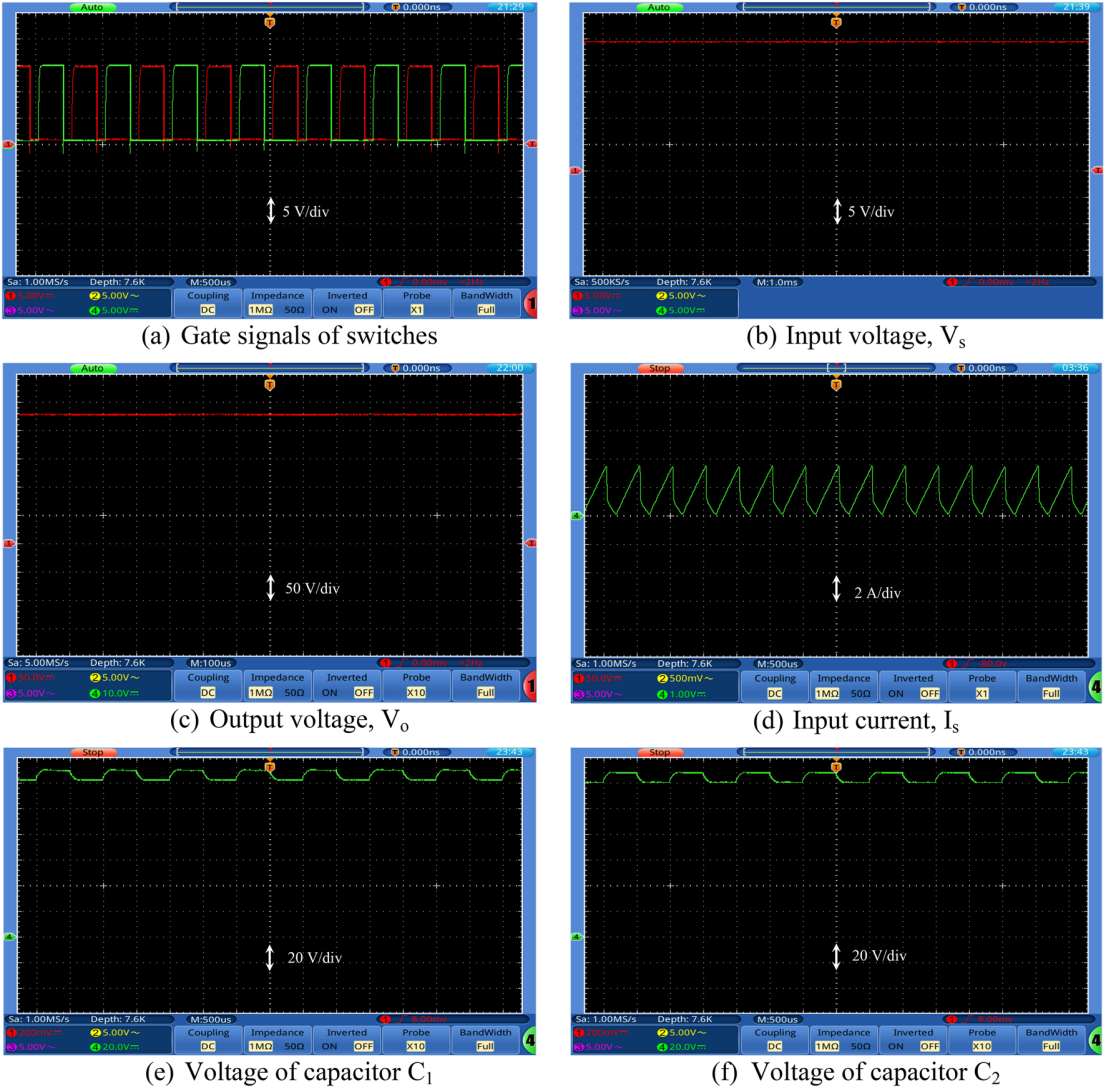
Experimental waveforms of the proposed converter. Part I. (**a**) Gate signals of switches, (**b**) Input voltage, V_s_, (**c**) Output voltage, V_o_, (**d**) Input current, Is, (**e**) Voltage of capacitor C_1_, and (**f**) Voltage of capacitor C_2_.

**Figure 8:**
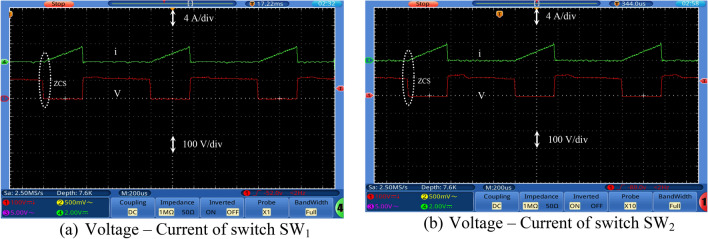
Experimental waveforms of the proposed converter. Part II. (**a**) Voltage–Current of switch SW_1_, and (**b**) Voltage–Current of switch SW_2_.

**Figure 9:**
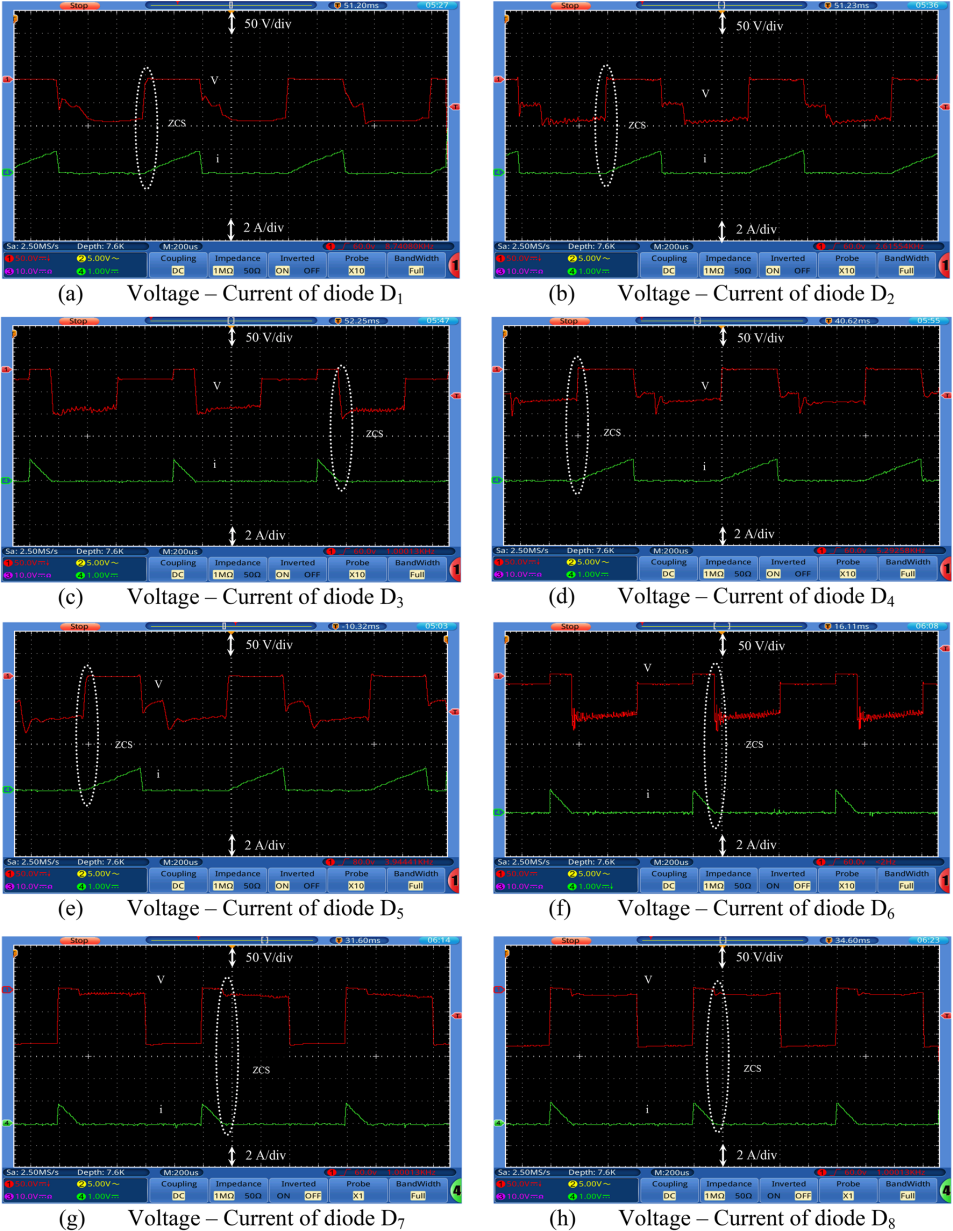
Experimental waveforms of the proposed converter. Part III. (**a**) Voltage–Current of diode D_1_, (**b**) Voltage–Current of diode D_2_, (**c**) Voltage–Current of diode D_3_, (**d**) Voltage–Current of diode D_4_, (**e**) Voltage–Current of diode D_5_, (**f**) Voltage–Current of diode D_6_, (**g**) Voltage–Current of diode D_7_, and (**f**) Voltage–Current of diode D_8_.

**Figure 10:**
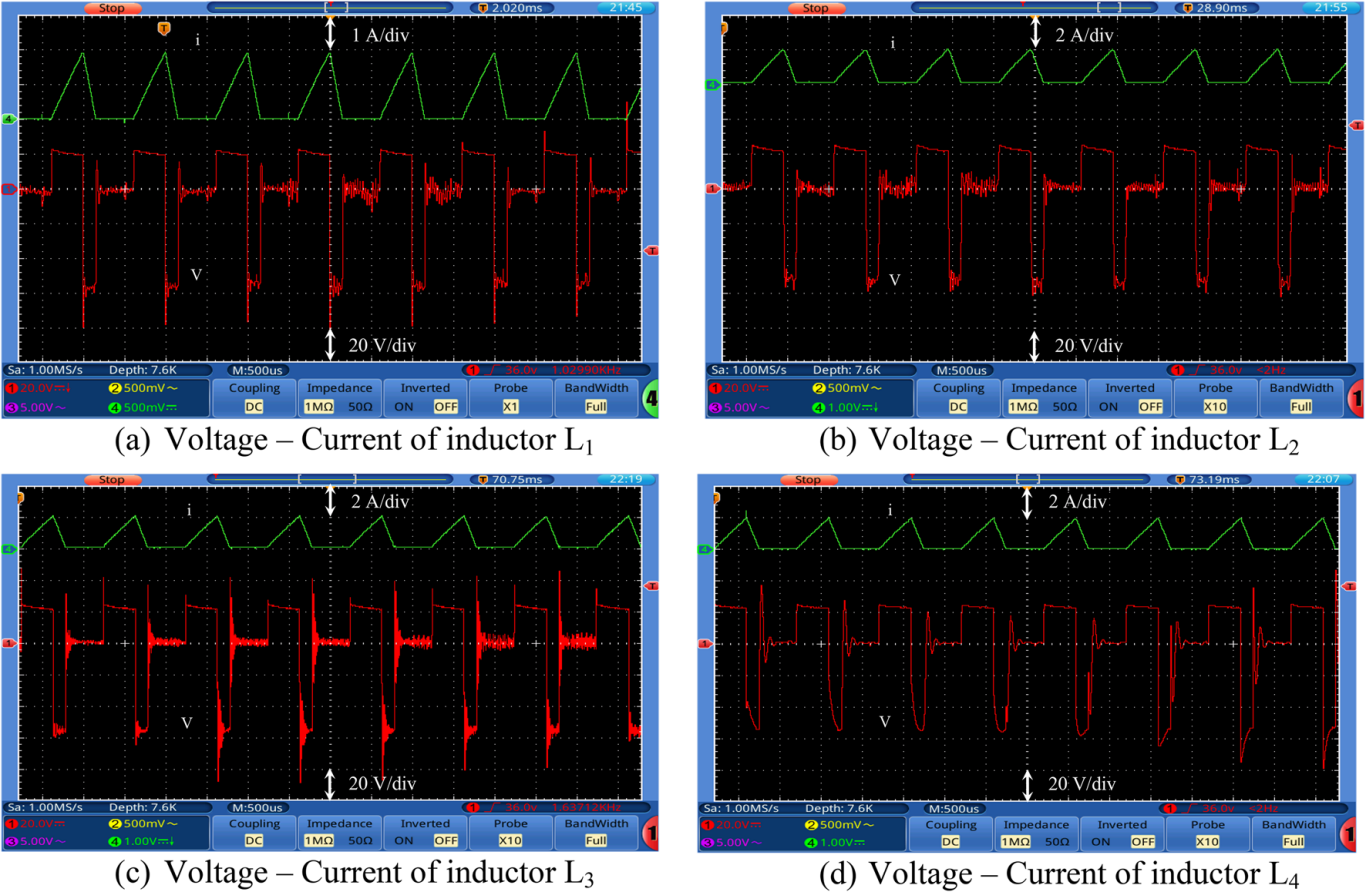
Experimental waveforms of the proposed converter. Part IV. (**a**) Voltage–Current of inductor L_1_, (**b**) Voltage–Current of inductor L_2_, (**c**) Voltage–Current of inductor L_3_, and (**d**) Voltage–Current of inductor L_4_.

Figures 7, 8, 9 and 10 show the experimental waveforms of the proposed converter at duty cycle 0.4. These figures include the resulted waveforms of the gate signal of switches SW_1_ and SW_2_, the input and output voltages, the input current, the voltage across the capacitors, and the voltage-current of the switches, diodes, and inductors. Figure 7a shows the gate pulses of SW_1_ and SW_2_. The input voltage supplied from PV is shown in Fig. 7b and equal to 24 V. It can be seen from Fig. 7c that the output voltage equals to 226 V which gives a gain of 9.4. This performance proves that the proposed converter gives a high voltage gain at reasonable duty cycle. Figure 7d shows the continuous input current waveform. As shown in Fig. 7e,f, both the voltage of the capacitors is equal and has an average value of 125 V that is equal to 55.3% of output voltage. That ensures a low voltage stress of the capacitors, and it also achieves the Eq. (7). As shown from Fig. 8a,b, the maximum voltage across each switch (SW_1_ or SW_2_) is almost equal to 57.5% of the output voltage which shows a low voltage stress for all switches. Furthermore, all switches operate in soft switching mode (ZCS) that decrease the switching losses of the switches. Moreover, from Fig. 9, the absolute maximum voltages across diodes *D*_1_, *D*_2_, *D*_3_, *D*_4_, *D*_5_, and *D*_6_ and across diodes *D*_7_ and *D*_8_ are almost equal to 39.8% and 53.1% of the output voltage, respectively which show a low voltage stress for all diodes. Also, all diodes operate in soft switching mode (ZCS) that decrease the diodes losses.

**Figure 11:**
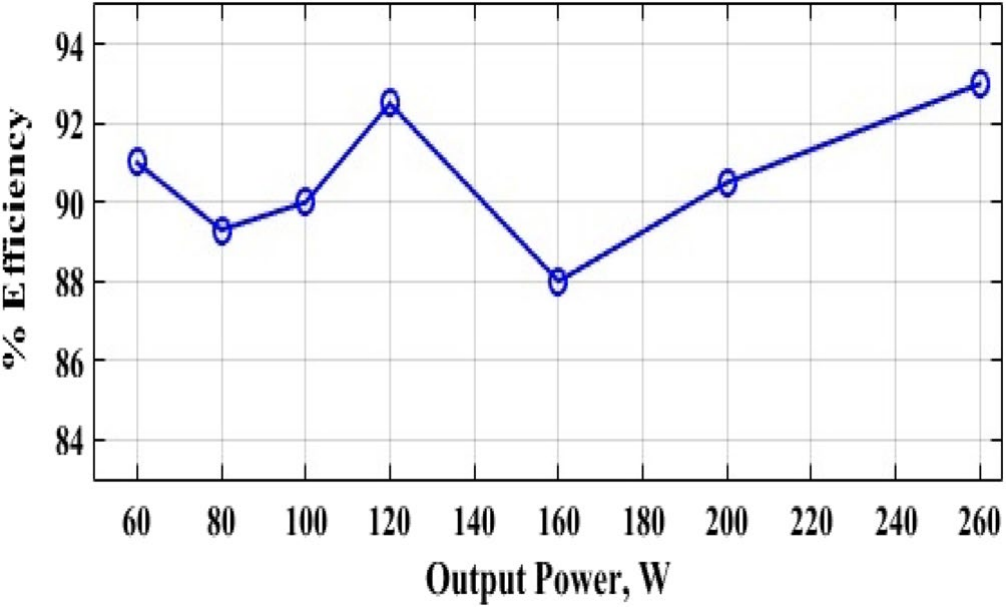
Experimental measured efficiency of the proposed converter.

**Figure 12:**
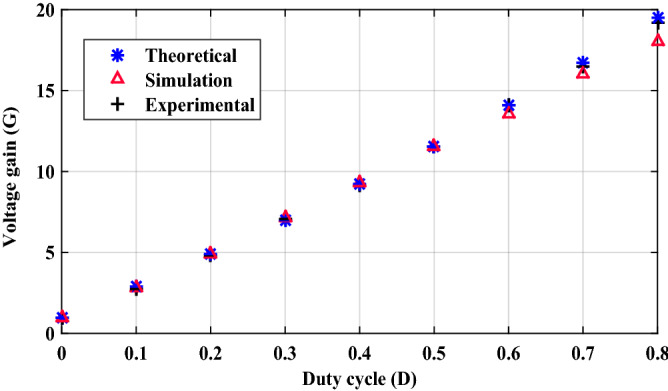
Voltage gain versus duty cycle of the proposed converter.

The inductor current (*i*_*L*_) is discontinuous as seen in Fig. 10 which shows that the converter is operating in DCM as mentioned before. Figure 11 shows the experimental measured efficiency of the proposed converter, in which it ascertains a high efficiency of the proposed topology. The maximum overall efficiency equals 93% measured at 120 W load. Figure 12 shows the voltage gain versus duty cycle of the theoretical equation, simulation results, and experimental results. It can be concluded that the experimental results are in good agreement with the theoretical analysis (Equation-14), and simulation results. From the previous experimental results, the proposed converter provides a high voltage gain with a low duty cycle, and it has small voltage stress of all semiconductor devices. Furthermore, a continuous input current is attained which a desirable feature of the DC/DC converter is making it suitable for PV applications. Therefore, it has low switching losses without any additional circuits. Also, it has components with a low nominal rating that makes the proposed converter small size, low price, and high overall efficiency. The aforementioned advantages make the proposed converter suitable for numerous industrial applications.

### Closed loop performance

**Figure 13:**
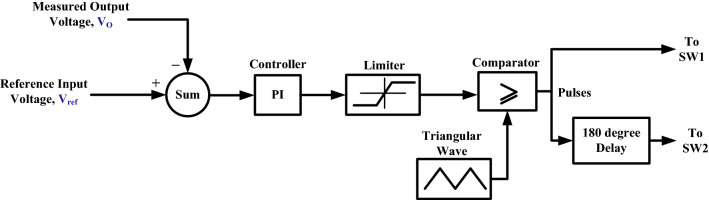
A schematic diagram of the control circuit used.

**Figure 14:**
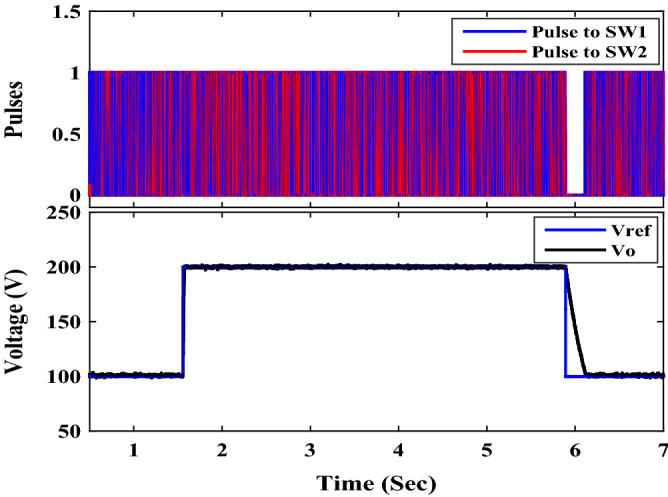
Experimental step change (increase/decrease) in reference voltage.

**Figure 15:**
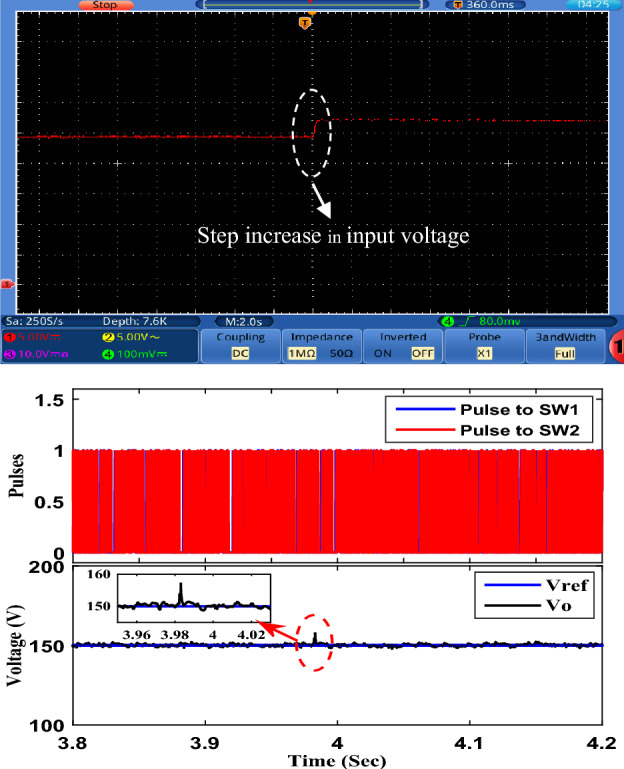
Experimental response to step increasing in input voltage.

**Figure 16:**
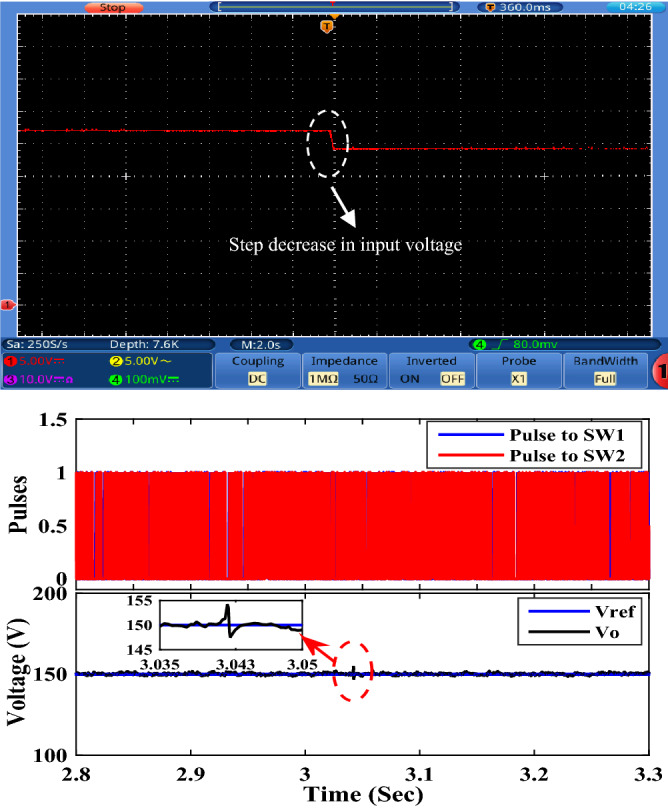
Experimental response to step decreasing in input voltage.

**Figure 17:**
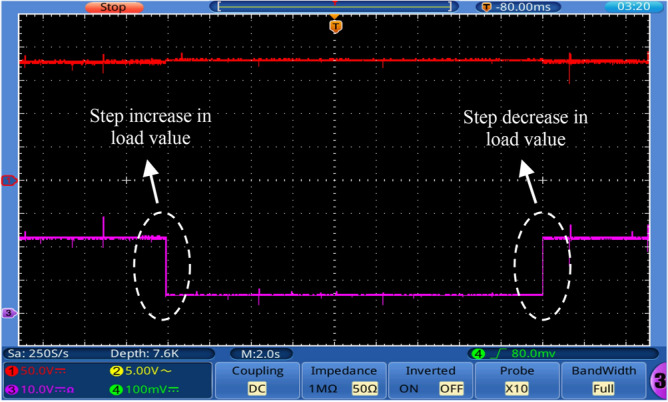
Experimental response to step change (increase/decrease) in load value.

To examine the performance of the proposed converter under closed-loop control, the OWON TDS8204 oscilloscope and *DSP*1104 are used to record the results. PI controller is also used for control. The parameters for the PI controller are *K*_*P*_ = 0.38 and *K*_*I*_ = 200. A schematic diagram of the circuit that is used to control the output voltage of the proposed circuit is shown in Fig. 13. First, the proposed converter is tested by changing the reference voltage (increased/decreased) by 100 V. It can be seen from Fig. 14 that the output voltage of the proposed converter responds to the step change (increase/decrease) of the reference voltage and changes from 100 to 200 V and then from 200 to 100 V according to the value of reference voltage. Also, the transient period is found very small and less than 200 ms. Second, the proposed converter is tested by changing the input voltage source (increase/decrease) by 4 V. It can be seen from Figs. 15 and 16 that the output voltage value is remaining constant at the reference voltage value of 150 V. Also, the overshoot value is less than 7.5 V, and it is equal to 5% of the output voltage. Finally, the proposed converter is tested for variation in load values. The response to the step change (increase/decrease) in load is shown in Fig. 17. The converter is operating at 65% of full load. At first, the load increased by 35% of full load value to make the converter operate at full load, and then the load decreased by 35% of full load value to make the converter operate at 65% of the full load again. It is evident from the figure that the proposed converter operates at constant output voltage under load changes.

### Comparison of the proposed converter with recent converters

**Figure 18:**
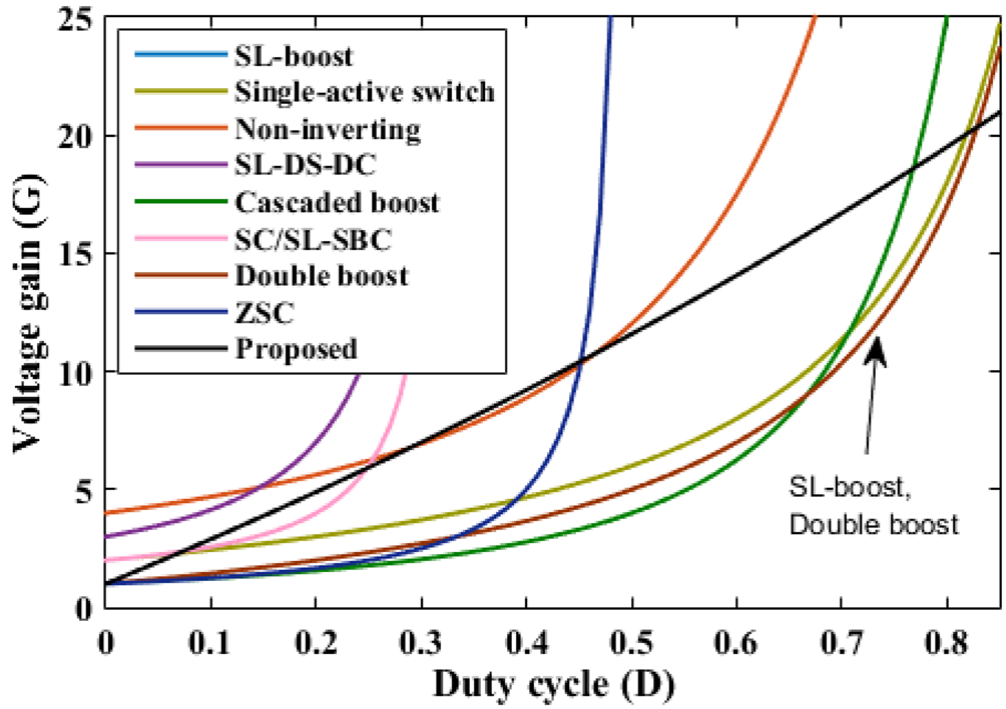
Voltage gain comparison.

**Figure 19:**
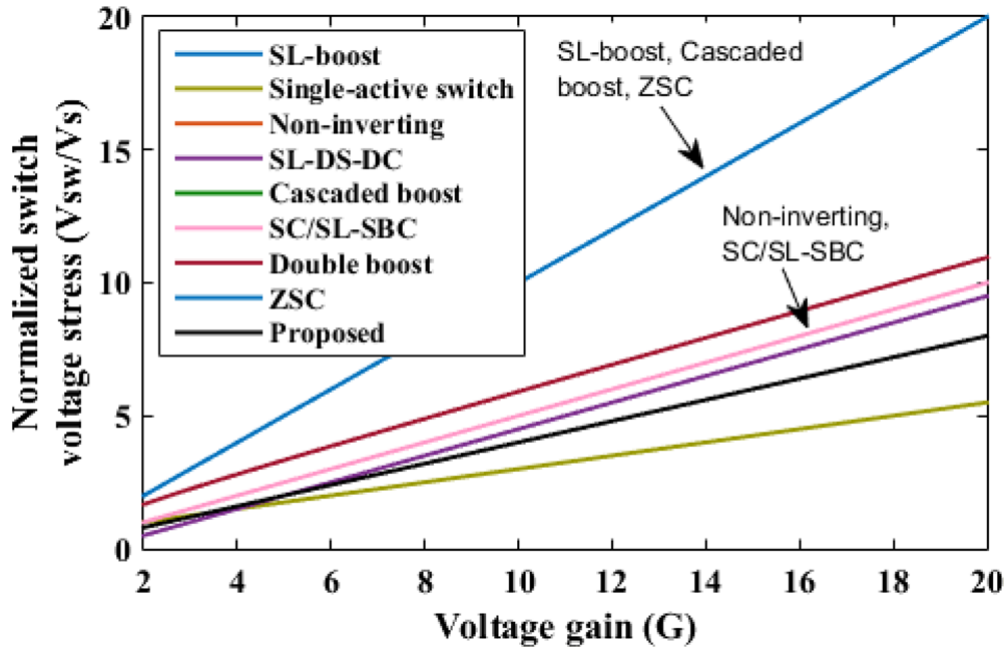
Switch voltage stress comparison.

**Figure 20:**
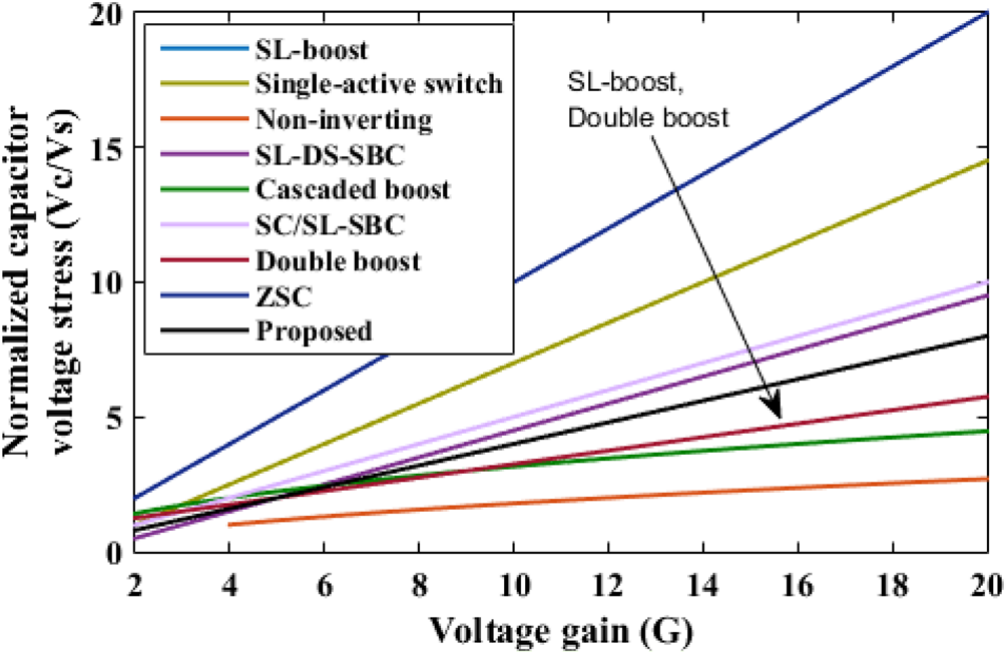
Capacitor voltage stress comparison.

**Figure 21:**
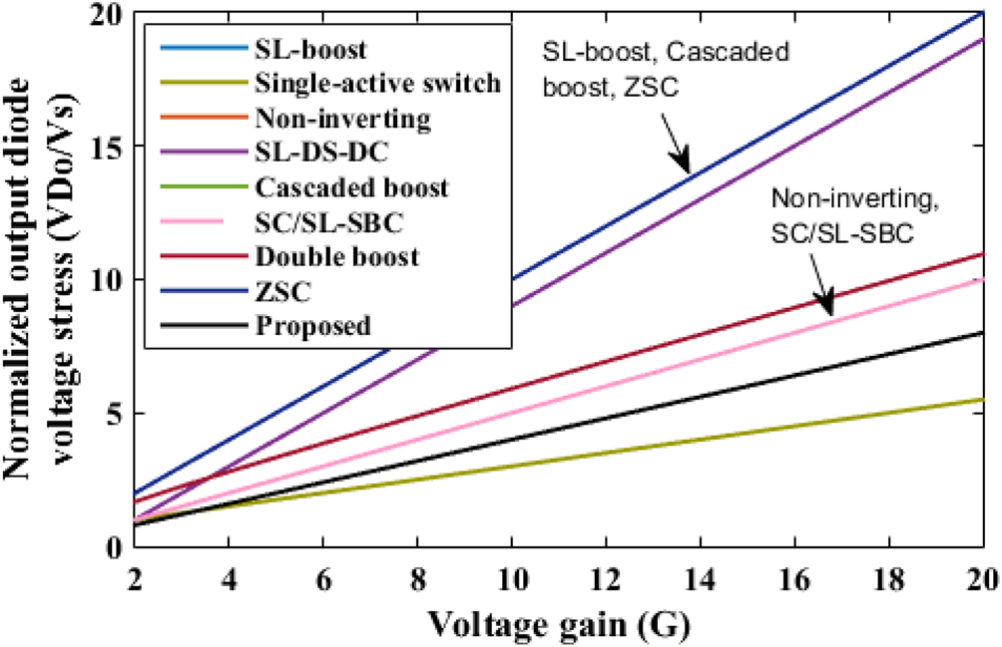
Output diode voltage stress comparison.

The proposed converter is compared with SL-Boost, single-active switch, non-inverting, SL-DS-DC, cascaded boost, SC/SL-SBC, double boost fly back, and ZSC converters. For a valid comparison, coupled inductor turns ratio (*n*) of the double boost-fly back converter was set to unity. The comparison results are presented in Table 2. The voltage gain comparison is presented in Fig. 18. It is clear that the proposed converter can operate with a wide range of duty cycle while the SL-DS-DC, and SC/SL-SBC converters operate only up to 0.3 duty cycle, and ZSC operates up to 0.5 duty cycle. Furthermore, the proposed converter has a higher gain at most of the duty cycle in comparison with SL-boost, single-active switch, cascaded boost, SC/SL-SBC, double boost fly back, and ZSC converters except SL-DS-DC converter that have the highest gain up to 0.3 only and the non-inverting converter. For the number of passive and active components, the proposed converter shows a modest number of active and passive components in comparison with SL-boost, single-active switch, non-inverting, SL-DS-DC, double boost fly back, and SC/L-SBC converters except cascaded boost and ZSC converters that have the lower components counts. However, they (cascaded boost and ZSC converters) operate at a larger duty cycle which may produce saturation problems in the inductor current or core. All the converters operate in continuous input current except the SL-boost, and ZSC converters. Also, the double boost fly back converter presents a discontinuous input current if it operates at a duty cycle of less than 0.5. The current ripple is low and within the allowed limit for all converters except the ZSC converter. Also, all converters operate with hard switching except the proposed converter that operates in soft switching for all semiconductor devices which makes lower losses and higher efficiency at higher gains. The switch voltage stress comparison is shown in Fig. 19. The proposed converter has a lower maximum switch voltage stress if it compared with all the converters except the single-active switch converter which has the smallest switch voltage stress. A lower switch voltage stress makes the losses lower and selecting a low nominal rating of switches that makes the converter small size, low price, and high overall efficiency. The capacitor voltage stress comparison is shown in Fig. 20. The proposed converter has a lower capacitor voltage stress if it compared with single-active switch, SL-DS-DC, SC/SL-SBC, and ZSC converters. A lower capacitor voltage stress gives a benefit of choosing a low nominal rating of capacitors that makes the converter smaller in size, and hence lower price. The output diode voltage stress is compared as shown in Fig. 21. The diode voltage stress of the proposed converter is lower than all the converters except the single-active switch converter. Lower voltage stress of output diode makes the nominal rating of diode much lower which affects the converter size and price. The efficiency of the proposed converter is reasonable compared to the other converters except for the cascaded boost converter which has the highest efficiency. However, the cascaded boost converter cannot accomplish a higher gain due to parasitic. Alternatively, a single-active switch converter has low voltage gain although it has similar elements count as the proposed converter. Theoretically, the converter power density depends on the number of semiconductor devices and the volume of the passive components. As known, the volume of passive components is proportional to the energy stored in them. So, if the stored energy is computed, then the volume of the passive components can be estimated. The total energy stored for the inductor is calculated by

$$ E_{L} = 0.5*L*({\text{i}}_{{L{ - }av}} )^{2} $$

where i_L-av_ is average current through the inductor *L*, and the total energy stored for the capacitor is given as

$$ {\text{E}}_{{\text{C}}} = 0.{5}*C*\left( {V_{{\text{C}}} } \right)^{{2}} $$

where *V*_C_ is voltage through the capacitor *C*.

**Table 2:** Comparison of proposed converter with recent converters.

	SL-boost^6^	Single-active switch^20^	Non-inverting^25^	SL-DS-DC^28^	Cascaded boost^29^	SC/SL-SBC^30^	Double boost flyback^32^	ZSC^33^	Proposed converter
Gain	$\frac{{1 + 3{\text{D}}}}{{1 - {\text{D}}}}$	$\frac{{2 + 2{\text{D}}}}{{1 - {\text{D}}}}$	$\frac{{2(2 - {\text{D}})}}{{(1 - {\text{D}})^{2} }}$	$\frac{{3 - {\text{D}}}}{{1 - 3{\text{D}}}}$	$\frac{1}{{(1 - {\text{D}})^{2} }}$	$\frac{{2(1 - {\text{D}})}}{{1 - 3{\text{D}}}}$	$\frac{{1 + 3{\text{D}}}}{{1 - {\text{D}}}}$	$\frac{1}{{1 - 2{\text{D}}}}$	$\frac{{1 + 18.25{\text{D}}}}{{1 - 0.25{\text{D}}}}$
Diodes	10	5	6	7	3	7	4	2	8
Inductors	4	3	2	2	2	2	4	2	4
Capacitors	1	7	5	3	2	3	4	3	2
Switches	1	1	1	2	1	2	2	1	2
Total device count	16	16	14	14	8	14	14	8	16
Continuous input current	No	Yes	Yes	Yes	Yes	Yes	For D > 0.5 only	No	Yes
Soft switching	No	No	No	No	No	No	no	No	Yes
Current ripple	Low	Low	Low	Low	Low	Low	Low for D > 0.5 only	High	Low
Switch voltage	Vo	Vo/(2 + 2D)	Vo/2	(Vo–VS)/2	Vo	Vo/2	Vo/(1 + D)	Vo	Vo/2.5
Capacitor voltage	$\frac{1}{{(1 - {\text{D}})}}{\text{V}}_{{\text{S}}}$	$\frac{{1 + 2{\text{D}}}}{{1 - {\text{D}}}}{\text{V}}_{{\text{S}}}$	$\frac{1}{{(1 - {\text{D}})}}{\text{V}}_{{\text{S}}}$	$\frac{{{\text{V}}_{{\text{o}}} - {\text{V}}_{{\text{S}}} }}{2}$	$\frac{1}{{(1 - {\text{D}})}}{\text{V}}_{{\text{S}}}$	$\frac{{{\text{V}}_{{\text{o}}} }}{2}$	$\frac{1}{{(1 - {\text{D}})}}{\text{V}}_{{\text{S}}}$	$\frac{1}{{1 - 2{\text{D}}}}{\text{V}}_{{\text{S}}}$	$\frac{{{\text{V}}_{{\text{o}}} }}{2.5}$
Diode voltage (Do)	Vo	Vo/(2 + 2D)	Vo/2	Vo–VS	Vo	Vo/2	Vo/(1 + D)	Vo	Vo/2.5
Ideal maximum duty cycle	1	1	1	0.3	1	0.3	1	0.5	1
Ideal gain at D = 0.2	2	3	5.6	7	1.7	4	2 at n = 1	1.7	4.9
Efficiency (%) at Pload = 200 W	93	93.2	89	90	94.8	80.3	93.1	90.5	90.4
Energy stored in inductor	8	10	12	10.5	20	48.7	7	60	8
Energy stored in capacitor	25	32	21	24	30	31.9	20	43	30

While evaluating the energy stored in the inductor, it is assumed that the frequency and ripple currents are the same for all the compared converters. Furthermore, the energy stored in the capacitor is computed for a similar value of capacitances.

For voltage gain, *G* = 5, the total energy stored in the inductor and capacitor for all the converters is recorded in Table 2. It is obvious that the total energy stored, i.e., volume required is modest in the proposed topology (38). The double boost flyback converter is the smallest (27), however it has minimal voltage gain. The quantity of energy stored in inductors and capacitors that mentioned in Table 2 is made to be unitless.

Based on the previous various performance parameters and characteristics comparison, the proposed converter gives a high voltage gain at a low duty cycle. It has a modest number of semiconductor devices with low voltage stress and hence small nominal voltage rating, and lower losses making the converter smaller in size, higher efficiency, and has a good performance. According to these comparisons, the proposed converter is considered a strong competitor to the other converters.

## Conclusion

In this paper, a new non-isolated high voltage gain DC/DC converter by integrating a dual boost converter with a switched inductor structure is proposed. The proposed converter operates with a modest duty cycle (less than 0.5) with a continuous input current. The converter operates with a soft switching (ZCS) for all diodes and switches which plays an important role in reducing the losses. A wide operating range of the duty cycle is available. An equal current sharing among boost inductors makes it easy to control. Also, the proposed converter offers high efficiency due to the low switching losses, lower voltage stress for all passive and active components, and the lack of reverse recovery loss on diodes. It requires a small inductor, and a small nominal rating for all semiconductor devices which reduces the size, weight, and price of the proposed converter. These features make the converter a good choice for many applications such as PV, x-ray, fuel cells, etc. Moreover, the description, operating modes in DCM, design guidelines, and open and closed-loop performance are presented. Besides, a comparative analysis with recent converters is presented. The converter is examined at various power ratings for efficiency analysis and maximum efficiency of 93% is achieved. Experimental results in open and closed-loop prove the good performance of the proposed converter.

## Data Availability

All data generated or analyzed during this study are included in this published article.

## References

[1] Ramanathan JGG, Urasak N (2022). Non-isolated interleaved hybrid boost converter for renewable energy applications. Energies.

[2] Paula W, Oliveria DS, Pereira DC, Tofoli FL (2015). ‘Survey on non-isolated high-voltage step-up dc-dc topologies based on boost converter’. IET Power Elect..

[3] Malik M, Ali A, Kumar D (2017). A two cascaded boost converter with high voltage gain module. Int. J. Comput. Electr. Eng..

[4] Karthikeyan V, Kumaravel S, Gurukumar G (2019). High step-up gain DC–DC converter with switched capacitor and regenerative boost configuration for solar PV applications. IEEE Trans. Circuits Syst. II Exp. Briefs.

[5] Kumar A, Wang Y, Pan X, Kamal S, Xiong X, Zhang RH, Bao D (2020). A high voltage gain DC–DC converter with common grounding for fuel cell vehicle. IEEE Trans. Veh. Technol..

[6] Axelrod B., Berkovich Y. & Ioinovici, A. Switched capacitor (SC)/switched inductor (SL) structures for getting hybrid step-down Cuk/Sepic/Zeta converters. In *IEEE International Symposium Circuits Systems, Kos, Greece*, 5063–5066 (2006).

[7] Axelrod B, Berkovich Y, Ioinovici A (2008). Switched capacitor/switched inductor structures for getting transformerless hybrid DC–DC PWM converters. IEEE Trans. Circuits Syst. Reg. Pap..

[8] Tewari N, Thazhathu S (2020). Family of modular, extendable and high gain dc–dc converter with switched inductor and switched capacitor cells. IET Power Elect..

[9] Kumar A, Wang Y, Pan X, Raghuram M, Singh SK, Xiong X, Tripathi AK (2020). Switched-LC based high gain converter with lower component count. IEEE Trans. Ind. Appl..

[10] Lu, J., Stegen, S. & Butler, D. High frequency and high-power density transformers for DC/DC converter used in solar PV system. In *IEEE 2nd International Symposium on Power Electronics for Distributed Generation Systems, Hefei, China* 481–484 (2010).

[11] Suryadevara R, Parsa L (2019). Full-bridge ZCS-converter-based high-gain modular DC–DC converter for PV integration with medium-voltage DC grids. IEEE Trans. Energy Conv..

[12] Chu GML, Lu DDC, Agelidis VG (2012). Flyback-based high step-up converter with reduced power processing stages. IET Power Electron..

[13] Joseph P, Devaraj E (2019). Design of hybrid forward boost converter for renewable energy powered electric vehicle charging applications. IET Power Electron..

[14] Bilsalam A, Boonyaroonate I, Chunkag V (2016). High-voltage gain zero-current switching push–pull resonant converter for small energy sources. IET Power Electron..

[15] Fan X, Sun H, Yuan Z, Li Z, Shi R, Ghadimi N (2020). High voltage gain DC/DC converter using coupled inductor and VM techniques. IEEE Access.

[16] Kumar A, Wang Y, Pan X, Xiong X, Reza MM, Al Jaafari K (2019). Modified a-source converter operating at lower voltage stress. IEEE Access.

[17] Shi ZH, Cheng KWE, Ho SL (2014). Static performance and parasitic analysis of tapped-inductor converters. IET Power Electron..

[18] Wai RJ, Duan RY (2005). High step-up converter with coupled inductor. IEEE Trans. Power Electron..

[19] Meinagh FAA, Yuan J, Yang Y (2020). Analysis and design of a high voltage-gain quasi-Z-source DC–DC converter. IET Power Electron..

[20] Mizani M, Ansari SA, Shoulaie A, Davidson JN, Foster MP (2021). ‘Single-active switch high-voltage gain DC–DC converter using a non-coupled inductor’. IET Power Electron..

[21] Eshkevari AL, Mosallanejad A, Sepasian MS (2021). Design, analysis, and implementation of a new high-gain P-type step-up dc/dc converter with continuous input current and common ground. IET Power Electron..

[22] Zhang G, Zhang B, Li Z, Qiu D, Yang L, Halang WA (2015). 3-Z-network boost converter. IEEE Trans. Ind. Electron..

[23] Zhang G, Iu HH-C, Zhang B, Li Z, Fernando T, Chen S-Z, Zhang Y (2017). An impedance network boost converter with a high-voltage gain. IEEE Trans. Power Electron..

[24] Abbasi M, Nazari Y, Abbasi E, Li L (2021). A new transformer-less step-up DC–DC converter with high voltage gain and reduced voltage stress on switched-capacitors and power switches for renewable energy source applications. IET Power Electron..

[25] Mahmood A, Zaid M, Ahmad J, Khan MA, Khan S, Sifat Z, Lin C, Sarwar A, Tariq M, Alamri B (2021). A non-inverting high gain DC–DC converter with continuous input current. IEEE Access..

[26] Khan S, Zaid M, Mahmood A, Nooruddin AS, Ahmad J, Alghaythi ML, Alamri B, Tariq M, Sarwar A, Lin C (2021). A new transformerless ultra high gain DC–DC converter for DC microgrid application. IEEE Access.

[27] Ghaffarpour M, Ebrahimi R, Kojabadi HM, Chang L, Guerrero JM (2021). Novel high voltage gain DC–DC converter with dynamic analysis. IET Power Electron..

[28] Bao D, Kumar A, Pan X, Xiong X, Beig AR, Singh SK (2021). Switched Inductor double switch high gain DC–DC converter for renewable applications. IEEE Access.

[29] Leyva-Ramos J, Diaz-Saldierna LH, Morales-Saldaæa JA, Ortiz-Lopez MG (2009). Switching regulator using a quadratic boost converter for wide DC conversion ratios. IET Power Elect..

[30] Zhu X, Zhang B, Li Z, Li H, Ran L (2017). Extended switched-boost DC–DC converters adopting switched-capacitor/switched-inductor cells for high step-up conversion. IEEE J. Emerg. Sel. Top. Power Electron..

[31] Zhao J, Chen D, Jiang J (2021). A novel transformerless high step-Up DC–DC converter with active switched-inductor and quasi-Z-source network. IET Power Electron..

[32] Cardos V, Junior SL, Lazzarin TB, Wattrich G (2020). Double boost—Fly back converter. IET Power Electron..

[33] Yang, L., Qiu, D., Zhang, B., Zhang, G. & Xiao, W. A modified Z-source DC–DC converter. In *IEEE 16th European Conference on Power Electronics and Applications, Lappeenranta* 1–9 (2014).

